# Correction: *Study protocol for a randomised controlled trial: harmonising optimal strategy for treatment of coronary artery stenosis — coronary intervention with next-generation drug-eluting stent platforms and abbreviated dual antiplatelet therapy (HOST-IDEA) trial*

**DOI:** 10.1136/bmjopen-2017-016617corr1

**Published:** 2018-02-10

Kim C, Han J, Yang H, *et al*. Study protocol for a randomised controlled trial: harmonising optimal strategy for treatment of coronary artery stenosis — coronary intervention with next-generation drug-eluting stent platforms and abbreviated dual antiplatelet therapy (HOST-IDEA) trial. *BMJ Open* 2017;**7:**e016617. doi: 10.1136/bmjopen-2017-016617

The author name ‘Kyu-Rock Han’ should be spelled ‘Kyoo-Rok Han’.

