# Supplementary material for: Study protocol for a randomised controlled trial: harmonising optimal strategy for treatment of coronary artery stenosis — coronary intervention with next-generation drug-eluting stent platforms and abbreviated dual antiplatelet therapy (HOST-IDEA) trial
Source: BMJ Open. 2017 Oct 11;7(10):e016617. doi: 10.1136/bmjopen-2017-016617 (PMC5652478; doi:10.1136/bmjopen-2017-016617)
Supplement: Supplementary appendix [file bmjopen-2017-016617supp001.pdf]

Appendix

Endpoint definitions

To gauge the clinical efficacy of each stent system, TLF as a composite of cardiac death, TVMI, and clinically driven TLR will be used as a surrogate for outcome measure. Though 1-year TLF rate will be presented as a primary endpoint of this trial, TLF and its components will be collected up to 3 years to delineate long-term clinical outcome. At the same time, NACE as a comprehensive outcome related to safety issue will be verified throughout the study period and it will disclose the consequences according to the two schemes of mandatory DAPT regimen. Definite or probable stent thrombosis and major bleeding as well as TLF will be regarded as components of NACE. Outcome measures and their components in this trial will follow the current data standards and regulatory guidelines [10 11].

1. Target lesion failure (TLF)

   The composite of clinically driven TLR, MI or cardiac death related to the target vessel. Target lesion is the segment where coronary intervention was performed, and the length of the target lesion is inclusive of the arterial segment treated with the stent and the 5mm proximal and 5mm distal to the treated section. Target vessel is the major native coronary artery or bypass graft containing the target lesion. A native coronary artery target vessel includes the arterial segments upstream and downstream from the target lesion and their major side branches. If it cannot be determined with certainty whether an MI or death is related to the target vessel, and at the same time if no other specific reasons can be given, it will be considered as a case of TLF.

2. Cardiac death

   Death by any cardiovascular mechanisms (arrhythmia, sudden death, low cardiac output heart failure, stroke, pulmonary embolism, or peripheral artery disease) will be

counted as a cardiac death. Unwitnessed death in a subject seen alive and clinically stable less than 24 hours before being found dead without any evidence supporting a specific non-cardiovascular cause of death will be also judged as a cardiac death. In addition, death caused by the immediate complications of the procedure will be managed as a cardiac death. Any death not covered by the above definitions, including death due to natural progression of underlying chronic disease, infection, accident, suicide or trauma will be handled as a non-cardiovascular death.

3. Target vessel myocardial infarction (TVMI)

   Among the case of acute spontaneous MI, TVMI is defined as a MI case with the evidence of myocardial necrosis in the vascular territory of previously treated target vessel. As well as direct evidence of invasive angiography, electrocardiographic or other imaging evidences such as echocardiography (e.g., newly developed regional wall motion abnormality or extension of previous abnormality) can be used to adjudicate the involvement of target vessel territory. Any types of MI related to stent thrombosis or restenosis of the target lesion will be included to TVMI case, but periprocedural MI (e.g., type 4a MI associated with and occurring within 48 hours of coronary intervention) and death with symptoms suggestive of myocardial ischemia but without direct evidence of target vessel involvement will be excluded from the outcome measure of TVMI.

4. Clinically driven target lesion revascularization (TLR)

   TLR indicates a revascularization procedure with repeated stenting, balloon angioplasty or surgical bypass grafting for restenosed or occluded culprit target lesion. TLR is clinically driven if the target lesion diameter stenosis is more than 50% by quantitative coronary angiography (QCA) and the subject has clinical or functional

ischemia that cannot be explained by another native coronary or bypass graft lesion. Even in the absence of ischemic symptoms or signs, TLR for a stented lesion with more than 70% diameter stenosis may be also considered clinically driven. And in case of the absence of QCA data or if a stenosis $\leq$50% is present, TLR may be considered clinically driven if severe ischemic signs and symptoms attributable to the target lesions are present. Meanwhile, repeated intervention or surgical bypass for any segment of a coronary artery containing a target lesion will be counted as a case of target vessel revascularization (TVR), and of course, TLR can be considered as TVR. In this context, target vessel failure (TVF) is defined as a composite of clinically driven TVR, cardiac death and TVMI, and this ancillary measure also will be counted throughout the study period.

5. Definite or probable stent thrombosis (ST)

Definite ST is defined as occurring when clinical presentation is consistent with acute coronary syndrome, and angiographic or pathologic examination with autopsy confirm stent occlusion or thrombus in the stented segment. Angiographic confirmation is the presence of a thrombus at the time of angiography that originates in or from the stent or in the segment 5mm proximal or distal to the stent. Probable ST is defined as any unexplained death occurring within the first 30 days that cannot be attributed to other causes. And irrespective of the time after the index procedure, any MI that is related to the territory of the implanted stent without angiographic confirmation of ST can be regarded as probable ST in the absence of any other obvious cause. Timing of the detection of ST will follow to the ARC (Academic Research Consortium) grading criteria: 0-24 hours after stent implantation is acute ST, >24 hours to 30 days after stenting is subacute ST, >30 days to 1 year after the procedure is late ST, and more than

1 year after stent implantation is very late ST.

6. Major bleeding

   Type 3 or 5 bleeding according to the BARC (Bleeding Academic Research Consortium) criteria will be counted as a major bleeding event. Overt bleeding with hemoglobin drop more than 3 g/dL, any transfusion, procedure-related cardiac tamponade, bleeding requiring surgical intervention or intravenous vasoactive agents and intracranial or intraocular hemorrhage will be recorded as a major bleeding event. Fatal bleeding and subsequent death without no other explainable cause also can be categorized into this class of bleeding.

Stent platforms for this trial

Orsiro™ stent system	
Stent backbone	PRO-Kinetic Energy® stent system
Stent alloy	Cobalt chromium alloy
Passive coating	PROBIO™ amorphous silicon carbide coating
Active coating	BIOlute™ high molecular weight Poly-L-Lactic Acid (PLLA)
Coating dose of sirolimus	1.4μg/mm^2
Stent strut thickness	Nominal diameter 2.25~3.00mm: 60μm (0.0024”)<br>3.50~4.00mm: 80μm (0.0031”)
Nominal diameter	2.25 / 2.50 / 2.75 / 3.00 / 3.50 / 4.00mm
Nominal length	9 / 13 / 15 / 18 / 22 / 26 / 30 / 35 / 40mm
Lesion entry profile	0.017” (0.43mm)
Lesion crossing profile	0.039” (0.99 mm)
Nominal inflation pressure	8 atm
Rate burst pressure	16 atm

Coroflex ISAR™ stent system	
Stent backbone	CX Blue Ultra & Neo® stent system
Stent alloy	Cobalt chromium alloy
Coating dose of sirolimus	1.2μg/mm^2
Stent strut thickness	Nominal diameter 2.00~2.50mm (): 50μm (0.0020”)<br>2.75~4.00mm (CX-Blue Neo): 60μm (0.0024”)
Nominal diameter	2.00 / 2.25 / 2.50 / 2.75 / 3.00 / 3.50 / 4.00mm
Nominal length	2.00~2.50mm stent:

	9 / 14 / 16 / 19 / 24 / 27 / 32mm<br>2.75~4.00mm stent:<br>8 / 13 / 16 / 19 / 24 / 27 / 32mm
Lesion entry profile	0.016” (0.41mm)
Lesion crossing profile	0.031~0.037” (0.79~0.93mm)
Nominal inflation pressure	10 atm
Rate burst pressure	18 atm (15 atm in case of 4.00mm-sized stent)
