# Supplementary material for: Study protocol for a randomised controlled trial: harmonising optimal strategy for treatment of coronary artery stenosis — coronary intervention with next-generation drug-eluting stent platforms and abbreviated dual antiplatelet therapy (HOST-IDEA) trial
Source: BMJ Open. 2017 Oct 11;7(10):e016617. doi: 10.1136/bmjopen-2017-016617 (PMC5652478; doi:10.1136/bmjopen-2017-016617)
Supplement: Author's manuscript [file bmjopen-2017-016617.draft_revisions.pdf]

BMJ Open

Study protocol for a randomized controlled trial: Harmonizing Optimal Strategy for Treatment of coronary artery stenosis – coronary Intervention with next generation Drug-Eluting stent platforms and Abbreviated dual antiplatelet therapy (HOST-IDEA) trial

Journal:	*BMJ Open*
Manuscript ID	bmjopen-2017-016617
Article Type:	Protocol
Date Submitted by the Author:	27-Feb-2017
Complete List of Authors:	Kim, Chi-Hoon; Seoul National University Hospital<br>Han, Jung-Kyu; Seoul National University Hospital<br>Yang, Han-Mo; Seoul National University Hospital<br>Park, Kyung Woo; Seoul National University Hospital<br>Lee, Hae-Young; Seoul National University Hospital<br>Kang, Hyun-Jae; Seoul National University Hospital<br>Koo, Bon-Kwon; Seoul National University Hospital<br>Lee, Namho; Hallym University Kangnam Sacred Heart Hospital<br>Cha, Tae-Joon; Kosin University Gospel Hospital<br>Yang, Tae-Hyun; Inje University Busan Paik Hospital<br>Jeong, Myung-Ho; Chonnam National University Hospital<br>Yoon, Myeong-Ho; Ajou University Hospital<br>Lee, Seung Uk; Kwangju Christian Hospital<br>Lee, Seung Jin; SoonChunHyang University Cheonan Hospital<br>Shin, Eun-Seok; Ulsan University Hospital<br>Kim, Jin Won; Korea University Guro Hospital<br>Cho, Jin-Man; Kyung Hee University Hospital at Gangdong<br>Han, Kyu-Rock; Hallym University Kangdong Sacred Heart Hospital<br>Pyun, Wook Bum; Ewha Womans University Medical Center Mokdong Hospital<br>kim, hyosoo; Seoul National University Hospital
**Primary Subject Heading**:	Cardiovascular medicine
Secondary Subject Heading:	Cardiovascular medicine
Keywords:	Stent, Polymer, Antiplatelet therapy

SCHOLARONE™
Manuscripts

Study protocol for a randomized controlled trial: Harmonizing Optimal Strategy for Treatment of coronary artery stenosis – coronary Intervention with next generation Drug-Eluting stent platforms and Abbreviated dual antiplatelet therapy (HOST-IDEA) trial

Chi-Hoon Kim,[1*] Jung-Kyu Han,[1*] Han-Mo Yang,[1] Kyung Woo Park,[1] Hae-Young Lee,[1] Hyun-Jae Kang,[1] Bon-Kwon Koo,[1] Namho Lee,[2] Tae-Joon Cha,[3] Tae-Hyun Yang,[4] Myung-Ho Jeong,[5] Myeong-Ho Yoon,[6] Seung Uk Lee,[7] Seung Jin Lee,[8] Eun-Seok Shin,[9] Jin Won Kim,[10] Jin-Man Cho,[11] Kyu-Rock Han, [12] Wook Bum Pyun,[13] Hyo-Soo Kim[1]

1. Seoul National University Hospital, Seoul, Korea
2. Hallym University Kangnam Sacred Heart Hospital, Seoul, Korea
3. Kosin University Gospel Hospital, Busan, Korea
4. Inje University Busan Paik Hospital, Busan, Korea
5. Chonnam National University Hospital, Gwangju, Korea
6. Ajou University Hospital, Suwon, Korea
7. Kwangju Christian Hospital, Gwangju, Korea
8. SoonChunHyang University Cheonan Hospital, Cheonan, Korea
9. Ulsan University Hospital, Ulsan, Korea
10. Korea University Guro Hospital, Seoul, Korea
11. Kyung Hee University Hospital at Gangdong, Seoul, Korea

For peer review only

12. Hallym University Kangdong Sacred Heart Hospital, Seoul, Korea

13. Ewha Womans University Medical Center Mokdong Hospital, Seoul, Korea

Correspondence to:

Hyo-Soo Kim, MD, PhD

Phone: 82-2-2072-2226; Fax: 82-2-766-8904; E-mail: usahyosoo@gmail.com

* These two authors equally contributed to this work.

* E-mail addresses of co-authors

Chi-Hoon Kim: lightcross@gmail.com

Jung-Kyu Han: hpcrates@gmail.com

Han-Mo Yang: hanname@gmail.com

Kyung Woo Park: kwparkmd@snu.ac.kr

Hae-Young Lee: hylee612@gmail.com

Hyun-Jae Kang: nowkang@snu.ac.kr

Bon-Kwon Koo: bkkoo@snu.ac.kr

Namho Lee: namholee@hallym.or.kr

For peer review only

Tae-Joon Cha: chatjn@gmail.com

Tae-Hyun Yang: yangthmd@naver.com

Myung-Ho Jeong: mhjeong@chonnam.ac.kr

Myeong-Ho Yoon: yoonmh65@hanmail.net

Seung Uk Lee: cardiosu@hanmail.net

Seung Jin Lee: drlsj3@gmail.com

Eun-Seok Shin: sesim1989@gmail.com

Jin Won Kim: kjwmm@korea.ac.kr

Jin-Man Cho: aceri@medimail.co.kr

Kyu-Rock Han: krheart@hallym.or.kr

Wook Bum Pyun: pwb423@ewha.ac.kr

Hyo-Soo Kim: usahyosoo@gmail.com

For peer review only

Abstract

Introduction

We have recently seen the introduction of newer-generation drug-eluting stents (DESs) with ultrathin struts that utilize advanced polymer technologies. However, the efficacy and safety of these newest stents have not yet been fully explored. In addition, there are still controversies over the optimal duration of dual antiplatelet therapy (DAPT) after stent implantation, particularly for ultrathin stents with the newest polymer technologies.

Methods and analysis

The HOST-IDEA trial is a randomized, open-label, multicenter, non-inferiority trial, and the first study to directly compare 2 of these ultrathin sirolimus-eluting stents (SES): Orsiro stent with biodegradable polymer, and polymer-free Coroflex ISAR (CX-ISAR) stent. This study has a scheme of 2×2 factorial design according to the stent type and DAPT duration (3- vs. 12-months). A total of 2,152 patients will be randomized and stratified to demonstrate the non-inferiority of CX-ISAR to Orsiro, or of the abbreviated DAPT duration to the conventional 12 months (both in 1:1 ratio). For the comparison of stent type, primary endpoint is target lesion failure (TLF) which is a composite of cardiac death, target vessel-related myocardial infarction, and clinically driven target lesion revascularization. For the comparison of DAPT duration, net adverse clinical event is the co-primary endpoint which is defined as a composite of TLF, definite/probable stent thrombosis and major bleeding.

Ethic approval and dissemination

All the institutions involved in this study are required to have ethical approval prior to patient enrollment. This multicenter study will recruit patients through competitive registration, but institutions that have not yet obtained ethical approvals have made it impossible to enroll

For peer review only

patients in a centralized Web database. The final results will be presented at relevant international conferences and will be materialized in the form of papers.

Trial registration number

NCT02601157; Pre-results.

Keywords

Stent, Polymer, Antiplatelet therapy

For peer review only

Strengths and limitations of this study

1. To the best of our knowledge, the clinical outcome of two up-to-date coronary stents with ultrathin strut, the Orsiro and Coroflex ISAR stents will be firstly compared in this randomized clinical trial. These two stents are based on the latest drug coating technology, however, no randomized studies have been reported to directly compare these two stents.
2. We could derive a meaningful result on the optimal duration of dual antiplatelet therapy (DAPT) in stents using ultrathin strut by adopting a 2x2 factorial design with a difference in the duration of DAPT maintenance (3- vs. 12-month). This study will confirm that the clinical performance is not worse than the conventional 12-month maintenance even if the DAPT maintenance period is kept short in the latest stents with thin strut thickness.
3. We also will be able to simultaneously test the difference between two co-primary outcomes, target lesion failure and net adverse clinical outcome, since we will register sufficient numbers of patients to secure statistical power.
4. To minimize potential risks and ensure patient safety, patients with ST-segment myocardial infarction (STEMI) who are generally recommended to apply a DAPT maintenance period of one year or more will be excluded from this study.

For peer review only

Introduction

Second generation drug-eluting stents (DESs) has been introduced to overcome the limitations of earlier versions of DESs such as late stent thrombosis and late catch-up.[1] The improvements of second generation DESs were made in many different fields as follows: better stent design with greater conformability, thinner strut thickness by using of new metal alloy, optimal load and improved release kinetics of drugs, and new polymer technology. All these improvements made second generation DESs safer and more efficacious.[2 3]

Thin strut thickness makes greater conformability, better deliverability, and lesser injury. This creates lesser shear disturbances, and reduces peri-strut inflammation and fibrin deposition, finally contributing to improved re-endothelialization.[4] Currently, the Orsiro hybrid sirolimus-eluting stent (SES) (Orsiro, Biotronik AG, Bülach, Switzerland) and the Coroflex ISAR (CX-ISAR, B. Braun Melsungen AG, Berlin, Germany) SES are two commercially available stents with thinnest strut thickness (60μm for diameter ≤3.0mm, 80 μm for diameter ≥3.5mm for Orsiro, 50μm for diameter ≤2.5mm, 60 μm for diameter ≥2.75mm for CX-ISAR). Interestingly, these two stent systems adopt two different up-to-date drug coating technologies. The Orsiro utilizes poly-L-lactic acid (PLLA) for biodegradable polymer.[5] While, sirolimus of the ISAR platform is coated on the stent strut without any polymer substance.[6]

The Orsiro stent showed non-inferior safety and efficacy outcomes compared with everolimus-eluting stents (EESs).[7] In contrast, previous version of the ISAR stent with stainless steel backbone was compared with zotarolimus-eluting stent (ZES), demonstrating its non-inferior safety and efficacy.[8] However, CX-ISAR, a latest version of the ISAR system with cobalt chromium (CoCr) alloy backbone, has not yet been tested in a large scale randomized controlled trial. Furthermore, up to now, there is no head-to-head comparison

For peer review only

between these two ultrathin stents with distinct drug eluting technologies, the Orsiro and CX-ISAR.

Meanwhile, optimal duration of dual antiplatelet therapy (DAPT) after DES implantation is still unconcluded. Current guidelines recommend 6-12 months of DAPT after DES implantation for patients with stable angina.[9] However, there are controversial studies regarding longer- versus shorter-duration DAPT. In particular, we have no data regarding optimal duration of DAPT for ultrathin stents with advanced polymer technologies. Because of potential of better re-endothelialization and less polymer-related adverse effects,[1 4] we can reasonably guess that shortened DAPT would be enough for these newer generation stents.

Therefore, this prospective, randomized, open-label, 2×2 factorial design multicenter trial was planned to address: (1) whether newly-developed ultrathin stents (Orsiro, CX-ISAR) are comparable to each other in terms of efficacy and safety, and (2) whether short duration of DAPT is non-inferior to conventional 1 year duration.

For peer review only

Methods and design

Study design and primary hypothesis

The HOST-IDEA trial is a randomized, open-label, multicenter, non-inferiority trial comparing the Orsiro with the CX-ISAR. The trial is powered to investigate a hypothesis that a polymer-free stent platform (CX-ISAR) is non-inferior to a biodegradable polymer-based stent (Orsiro) as regards post-procedure 1-year target lesion failure (TLF), as a composite of cardiac death, target vessel myocardial infarction (TVMI), and clinically driven target lesion revascularization (TLR). At the same time, another hypothesis will also be examined: 3 months' DAPT may deliver the same clinical efficacy and safety as conventional 1-year DAPT strategy. For this purpose, net adverse clinical events (NACE), defined as a composite of TLF, definite or probable stent thrombosis, and major bleeding according to the pertinent criteria,[10 11] will be checked as a co-primary endpoint. A 2×2 factorial design will be used to address these questions. Unless there is significant interaction between the two interventions, this factorial design can provide a useful scheme for testing two interventions simultaneously in a single dataset, and can be used to minimize sample size without limiting the statistical power.[12]

Study population and eligibility criteria

All participating centers are tertiary referral hospitals in Korea. Patients eligible for coronary intervention will be qualified with coronary angiography before the enrollment. Every participant will have at least 1 stenotic coronary lesion of >50% diameter stenosis suitable for stent implantation. To secure the statistical power and to obtain clear practical implications, high-risk patients for ischemic adverse events will be excluded in this protocol; patients with

For peer review only

ST-segment elevation myocardial infarction (STEMI) or unstable conditions such as cardiogenic shock or severe heart failure at the time of presentation. Detailed criteria for inclusion and exclusion are listed in Table 1.

Rationale for sample size calculation

Among the contemporary DESs, no stent platform has been directly compared to both Orsiro and CX-ISAR. And, the latest version of the ISAR system with a CoCr backbone, CX-ISAR, has not yet been tested in a large-scale randomized controlled trial (RCT). Instead, the Orsiro and previous versions of the ISAR system have been tested against EES and ZES, respectively, and clinical outcomes were found to be comparable in a number of large-scale studies.[7 8 13 14] The Orsiro was non-inferior to the Xience EES in the BIOSCIENCE trial,[7] and previous stainless steel-based ISAR platform and the Resolute ZES system had similar efficacy in the ISAR-TEST 5 trial.[8] As ZES and EES have similar efficacy in treating coronary disease,[15 16] it is reasonable to assume that the Orsiro and ISAR system will have similar levels of clinical efficacy and safety.

Other evidence supports this assumption. The TLF rate of Orsiro in the BIOSCIENCE trial was 6.5% over a 1-year follow-up.[7] In contrast, the ISAR system had a 1-year TLF rate of 13.1% in the ISAR-TEST 5 study.[8] This discrepancy was mainly due to the difference in TLR, rather than cardiac death or TVMI. The TLR rate of the ISAR system was somewhat higher than that of the Orsiro (1.9% cardiac deaths in the Orsiro vs. 1.9% in the ISAR system, TVMI 2.9% vs. 2.4%, TLR 4.0% vs. 10.3%, respectively). However, this contrast may be due to the study's policy regarding angiographic follow-up rather than the nature of the stent system itself. In the ISAR-TEST 5 trial, 6–8 months after the procedure, about three-quarters of patients (76.3%) had undergone dedicated angiographic surveillance,[8] whereas in the

For peer review only

BIOSCIENCE trial, angiography was performed at the time of 13-month follow-up. Consequently, 1-year clinical outcomes of the Orsiro could avoid the potential influence of routine angiographic follow-up, and this trial was able to minimize the risk of oculostenotic revascularization for non-ischemic intermediate lesions.[17] In a similar vein, 1-year TLR rate of 10.4% for the Resolute ZES system was also reported in the ISAR-TEST 5 trial, but in the Resolute all-comer study, without the impact of routine angiographic follow-up within 12 months, the same stent system had a lower TLR rate of 3.9%.[15]

Based on these data, it is reasonable to assume that the CX-ISAR and Orsiro would have similar TLR rates. Accordingly, 1-year TLF rate of the Orsiro in the BIOSCIENCE trial was employed as a reference for power calculation. With the assumption of a TLF rate of 6.5% and allowing for about 10% withdrawals or dropouts, a total of 2152 patients in 1:1 randomization will provide more than 80% power to detect a non-inferiority margin of 2.8% with a one-sided type I error of 0.05. These parameters are comparable to those of the BIOSCIENCE trial (reference value and non-inferiority margin 8.0% and 3.5%, respectively).[7] This size calculation may be able to secure the statistical power in case the event rates may be lower than expected. Non-inferiority margin of 2.5% for event rate of 5%, or non-inferior margin of 2.7% for event rate of 6% can be examined with this sample size even allowing for 10% withdrawal or dropouts.

This sample size might also be sufficient to test the second hypothesis. To date, no detailed data on the NACE rate of the CX-ISAR system have been reported. For the Orsiro stent, because most cases of stent thrombosis can also be counted as TLF events (cardiac death or MI), rates of 6.5% for TLF and 3.0% for major bleeding could be cited as references for 1-year NACE rate. Assuming patients with the Orsiro and 1-year DAPT have a NACE rate of 9.5%, 1039 patients will be required for each group of 3-months vs. 1-year DAPT to

For peer review only

distinguish a 3.2% margin of non-inferiority with an α value of 0.05 and 80% power. There is no actual interaction between the two interventions of randomization, maintenance duration of DAPT and allocated stent types. Therefore, even though the sample size of 2152 patients might be insufficient to tell the difference of individual components of NACE, this sample size might cover some patient loss and could provide enough statistical power to verify the second hypothesis simultaneously in a 2×2 factorial design. The 3.2% non-inferiority margin is similar to the reference values of the RESET and OPTIMIZE trials.[18 19]

Enrollment, procedure and study medications

After the identification of the target lesion and patients' consent to participate, randomization to stent type and DAPT duration will be performed using an electronic web-based database (© Cardiovascular Center, Seoul National University Hospital, and CRSCube Software, Seoul, South Korea) according to the 2×2 factorial design (Figure 1). Block randomization with block size of 8 and equal allocation probability for each group will be maintained throughout the entire study period. All the information about demographic, procedural, and follow-up data will be integrated into this centralized encrypted electronic database. Though this trial is an open-label study, these data will be managed by independent research nurses or other well-trained professionals.

Coronary intervention will be performed according to generally accepted current guidelines.[9 20] To improve applicability of this trial, DAPT regimen with prasugrel or ticagrelor as well as clopidogrel will be allowed. Every antiplatelet-naïve patient undergoing an elective procedure will be given 300 mg aspirin and loading dose of one of $P2Y_{12}$ receptor inhibitors (e.g., 600 mg clopidogrel, 60 mg prasugrel or 180 mg ticagrelor) preferably ≥2 hours before the intervention. These loading doses can be waived for chronic antiplatelet

For peer review only

users. Choice for $P2Y_{12}$ inhibitors will be based on the current guidelines as well as patient/lesional characteristics. To avoid possible bias, clopidogrel will be preferentially used for patients with stable angina whereas prasugrel or ticagrelor will be used mainly for patients with acute coronary syndrome.[9] Patients with higher bleeding risk such as patients older than 75 years of age, history of ischemic stroke or propensity to bleed can be treated with clopidogrel even in the case of acute coronary syndrome.

During the procedure, unfractionated heparin in a dose of at least 5000 IU or 70–100 IU/kg body weight will be administered for anticoagulation, while bail-out glycoprotein IIb/IIIa inhibitors, such as abciximab, will be left to the operators' discretion. Any lesional characteristics will be allowed for enrollment, except for in-stent restenosis of stented segments or previous treatment with balloon angioplasty. In addition to the angiographic findings, additional evaluations, such as intravascular ultrasound, optical coherence tomography (OCT), or fractional flow reserve assessed with pressure wire, may be used to characterize target lesions. Staged intervention can also be reserved for patients with complex lesional characteristics or multivessel disease, but even in these cases, target lesions can be treated only with the allocated stent platform. Because the Orsiro and CX-ISAR systems have similar configuration (see appendix), they are expected to be interchangeable, with no substantial differences in the procedure.

Follow-up and data collection

A DAPT schedule according to the web-based randomization is mandatory for every participant. Daily maintenance dose of aspirin is 100 mg and all patients will be given maintenance dose of $P2Y_{12}$ inhibitors according to their allocation (clopidogrel at a dose of 75 mg daily, prasugrel 10mg daily (5mg daily for patients with body weight of less than 60

For peer review only

kg) or ticagrelor 90mg twice a day). DAPT will be continued for up to 3 months or 1 year, as planned. To check patient's adherence to the DAPT regimen, a drug compliance survey will be conducted at the 1- and 3-month clinical follow-ups. Within this initial 3 months of follow-up, any unexpected discontinuation of DAPT will be regarded as a violation of the study protocol. After 3-month follow-up, brief interruptions of ≤5 days for elective surgery or planned procedures will be permitted, but every interruption of ≥6 days will be classified as non-adherence to the allocated 1-year DAPT regimen. This issue will be addressed for every case during the follow-up. Clinical follow-ups at the time of 1-, 3- and 12-month after the study enrollment are mandatory for the completion of the study. And during the period between 3 and 12 months, every patient will be required for out-patient visits at an interval less than 3 months. In addition to survey for DAPT compliance at the time of every out-patient visit, telephone interview will be given to patients who miss their scheduled appointments. DAPT with aspirin and clopidogrel/prasugrel/ticagrelor may be also extended beyond the predefined period, according to the patient's risk and the responsible clinician's discretion. Allocated $P2Y_{12}$ inhibitors will not be changed to other agents during the entire study period. But for patients with higher ischemic risk, prasugrel or ticagrelor may replace clopidogrel after the predefined period of DAPT maintenance.

Regardless of violation or drop-out, clinical outcomes as well as drug compliance up to 3 years will be collected in the centralized web database; data entry will be assigned to independent professionals. Any serious adverse events, including death, MI, revascularization, stent thrombosis, and bleeding will be entered into the web database for up to 3 years in a blinded fashion. These events will be adjudicated independently by a blinded adjudication committee. Central and on-site data monitoring will be performed according to a predefined monitoring plan. Every electronic case report form will be checked by central data monitoring. On-site monitoring will also be performed to secure data integrity; records of the

For peer review only

first 10 patients and subsequently a random 20% of the total registered patients will be verified. Dedicated angiographic surveillance will be scheduled at the time of 13-month follow-up, but this is not mandatory. Detailed instructions will be provided to each institution.

Statistical analysis

Interim analyses will be performed to test the feasibility of this trial when half of the enrolled patients have their 1-year results. Potential interactions between the stent types and the recommended maintenance duration of DAPT will be identified to ensure the statistical power of this trial, before the study hypotheses are addressed.[12] The primary outcome will be examined from an intention-to-treat viewpoint, but considering the potential influences of protocol violation or drop-out, per-protocol analysis will be used at the same time. The per-protocol population will be limited to patients with (1) a successful procedure treated solely with the allocated stent type, (2) no violation of recommended antiplatelet strategy, and (3) complete clinical follow-up information.

Using the proportional-hazards model, clinical outcomes will be compared between the stent types and DAPT strategies, possibly after controlling for relevant covariates. Stratified analyses for the primary endpoint across major subgroups will be performed using the Mantel–Cox method. Subgroup analyses will be stratified by male sex, smoking history, diabetes mellitus, chronic kidney disease of stage ≥3, off- versus on-label indication, small vessel (≤2.75 mm) or long target lesion (>28 mm), and complex lesion (type B2/C) or chronic total obstruction. Rates of bleeding complications will be analyzed according to the allocated antiplatelet strategy.

For peer review only

Definitions of outcome

Outcome measures and endpoint concepts will follow the definitions suggested in current recommendations.[11] The primary endpoint in this trial is TLF, as defined above, while the composite outcome of NACE will be managed as a co-primary endpoint. Detailed definitions are summarized in the appendix.

For peer review only

Discussion

The HOST-IDEA RCT is the first study to directly compare 2 ultrathin CoCr backbone stents with up-to-date polymer technologies: the sirolimus-eluting Orsiro stent with biodegradable polymer, and the sirolimus-eluting polymer-free CX-ISAR stent. These two stents are clearly distinct from other contemporary drug-eluting stents (DES) with durable polymers. Strut thickness is 50-60μm for Orsiro stent, and 60-80μm for CX-ISAR stent. Ultrathin strut thickness makes these stents more conformable and deliverable. In addition, injuries to the arterial wall during stent implantation and subsequent peri-strut inflammation can be minimized.[4] This feature may also contribute to more rapid arterial healing and more reliable endothelial coverage.[13 21] Further, these two stent platforms have adopted different up-to-date polymer technologies. Orsiro stent utilizes two-tiered hybrid polymer coating technology.[13 22] The outer layer is made of poly-L-lactic acid, which completely degrades over about 1 year period. The inner layer is silicon carbide inert matrix, which prevents the CoCr struts from being exposed to the diseased segment.[5] This unique hybrid system may greatly reduce chronic local inflammation around the stent struts and lessen the risk of denuded struts without re-endothelialization. In contrast, CX-ISAR utilizes polymer-free drug release technology. A mixture of sirolimus, probucol, and shellac resin is mounted into numerous micropores on the stent strut.[6 23] Since the sirolimus in this dual drug-delivery system has the same eluting profile to that of the lipophilic solvent probucol, controlled drug release is enabled for up to 6-8 weeks, and nothing will be left on the stent struts after 3 months.

Few previous studies assessed the clinical outcome of the Orsiro stent. The BIOSCIENCE trial randomized 1,063 patients to Orsiro and 1,056 patients to Xience EES stent.[7] Clinical efficacy of the Orsiro stent was comparable to that of EES, widely used durable polymer stent (1-year TLF rate in Orsiro 6.5% vs. 6.6% in EES group, P for non-inferiority <0.0004). The

For peer review only

safety profile of the Orsiro was also reliable: only 9 cases (0.9%) were reported as definite stent thrombosis during the 1-year follow-up period, compared to 4 cases (0.4%) in EES group (P = 0.16). Interestingly, in this trial, STEMI patients treated with the Orsiro were associated with a lower risk for 1-year TLF: 7 cases (3.3%) of 211 STEMI patients in Orsiro group vs. 17 (8.7%) of 196 in EES group (rate ratio 0.38, 95% CI 0.16–0.91, P for interaction 0.014). In contrast, the efficacy and safety of CX-ISAR have not been examined yet in a large-scale RCT. The HOST-IDEA RCT will provide the data regarding the efficacy and safety of the Orsiro and CX-ISAR by comparing these two stents.

In addition, there is still controversy over the optimal duration of DAPT following DES implantation. Several previous trials demonstrated that a shortened DAPT strategy was comparable to conventional 1 year strategy. The REAL-LATE/ZEST-LATE trials analyzed 2,701 patients who had received SES (57%), paclitaxel-eluting stent (PES) (24%), ZES (19%) or other DESs.[24] The results demonstrated that DAPT longer than 12 months was not more effective compared with aspirin monotherapy to reduce the rate of myocardial infarction (MI) or cardiac death. Our group previously compared 6-month to 12-month DAPT in 1,443 patients who underwent Xience/Promus EES or Cypher SES implantation in the EXCELLENT RCT.[25] We revealed that 6-month DAPT did not increase the risk of target vessel failure or the incidence of safety endpoint. And the RESET trial showed that 3-month DAPT following Endeavor ZES implantation was non-inferior to 12-month DAPT following other DES in 2,117 patients with respect to the occurrence of the primary endpoint consisting of cardiovascular death, MI, stent thrombosis, target/vessel revascularization, or bleeding.[18] In the PRODIGY trial, 2,013 patients who received bare-metal stent, PES or EES were randomly allocated to take 6-month or 24-month DAPT. As a result, a 24-month DAPT was not more effective than 6-month regimen in reducing the composite of all-cause mortality, MI or cerebrovascular accident, whereas there was a greater risk of bleeding in the 24-month

For peer review only

group.[26] In the OPTIMIZE trial including 3,119 patients after ZES implantation, 3-month DAPT was non-inferior to 12-month therapy for the primary endpoint composed of all-cause mortality, MI, stroke, or major bleeding.[19] In contrast, some recent trials demonstrated that prolonged DAPT significantly reduced thrombotic adverse clinical events. In the DAPT trial, 9,961 patients after 12-month DAPT following DES implantation were randomly assigned to continue DAPT or not. DAPT beyond 1 year after DES placement reduced the risks of stent thrombosis and major adverse cardiovascular and cerebrovascular events at the expense of an increased risk of bleeding.[27] The PEGASUS-TIMI 54 trial randomly allocated 21,162 patients with MI more than 1 year earlier to take an additional ticagrelor or placebo on top of aspirin. The results showed that prolonged DAPT using ticagrelor reduced the risk of cardiovascular death, MI, or stroke, but increased rates of major bleeding.[28]

Regarding this DAPT issue, the HOST-IDEA RCT also examines the safety and efficacy of the abbreviated DAPT duration in DAPT duration arm. Particularly, this trial will provide the first evidence regarding the optimal DAPT duration for the ultrathin stents with the newest polymer technologies.

For peer review only

Ethical approval and status of the trial

Ethics approval and consent to participate

At the time of submission (February 2017), a total of 13 centers are participating in this trial. This prospective trial had been approved from 8 centers, as of February 2017, name of the regulation authority and the issued IRB number are as follows: the review board of Seoul National University Hospital (D-1508-118-697), SoonChunHyang University Cheonan Hospital (SCHCA 2016-03-012), Hallym University Kangnam Sacred Heart Hospital (2016-04-31), Korea University Guro Hospital (MD16043), Ajou University Hospital (AJIRB-MED-DE4-16-170), Chonnam National University Hospital (CNUH-2016-096), Kwangju Christian Hospital (KCH-D-2016-03-003), Inje University Busan Paik Hospital (16-0117). Other 5 centers are on the review process with the same protocol and consent form (Kosin University Gospel Hospital, Ulsan University Hospital, Kyung Hee University Hospital at Gangdong, Ewha Womans University Medical Center Mokdong Hospital, Hallym University Kangdong Sacred Heart Hospital). Recruitment will not begin in any of these 5 centers until all local approvals have been obtained. The steering committee of this trial takes the responsibility for the study design. 13 independent regulation authorities of this trial are in full compliance with Good Clinical Practice as defined under the Korean Ministry of Food and Drug Safety regulations and the International Conference on Harmonization guidelines.

All patients will receive sufficient information to make a decision about participation before providing their written informed consent. Informed consent will be obtained by independent research nurses of well-trained personnel of each participating center. And every participant will have the right to withdraw their consent without restriction. Deferred consent will not be permitted for this study. Consent to publication will be obtained as a part of the general consent form, and individual patient data will be processed anonymously.

For peer review only

Trial registration and current status of this trial

This trial was registered at clinicaltrials.gov (NCT02601157; November 7, 2015). On January 28, 2016, we enrolled patients for the first time at the coordinating center (Seoul National University Hospital), since then 6 participating centers have begun to register patients. A total of 143 patients had been enrolled as of February 2017, and we expect patient registration will be extended to other institutions by the end of this year. This paper translates the study protocol version 1.0. Any protocol amendments or revisions will be communicated with researchers involved in this trial and mentioned in the results paper.

Availability of data and material

This article is distributed under the terms of the Creative Commons Attribution 4.0 International License (http://creativecommons.org/licenses/by/4.0/), which permits unrestricted use, distribution, and reproduction in any medium, provided you give appropriate credit to the original author(s) and the source, provide a link to the Creative Commons license, and indicate if changes were made. The Creative Commons Public Domain Dedication waiver (http://creativecommons.org/publicdomain/zero/1.0/) applies to the data made available in this article, unless otherwise stated. But, study results and conclusion of this trial will be separately covered in the results paper, and the dataset of this trial will not be shared unless otherwise stated.

Funding

This study was supported by the Korea Health Technology R&D Project (the grant from the

For peer review only

‘Korea research-driven hospital’ project, HI14C1277) through the Korea Health Industry Development Institute (KHIDI), funded by the Ministry of Health & Welfare (MHW). And, this study is an investigator-initiated trial which is partly supported by an unrestricted grant from Biotronik Korea (Seongnam-si, Gyeonggi-do, South Korea) and B.Braun Korea (Seoul, South Korea), . These two companies equally contributed to this study, but funding source did not involve in the study design. And they will have no role in the data collection and management. Data analysis, its interpretation and publication will be done without any interference.

Competing interests

All researchers, including the chair of this study – Hyo-Soo Kim, M.D., PhD., have no conflict of interest.

Authors’ contributions

Dr. H-S Kim, as a corresponding author, proposed the original concept and idea for this HOST-IDEA trial and supervised overall process of preparation. C-H Kim and J-K Han made a draft of the study protocol and prepared this manuscript (These two authors equally contributed this paper.) Patient consent form and centralized web-based database were prepared by C-H Kim. H-M Yang, K W Park, H-Y Lee, H-J Kang and B-K Koo critically reviewed the study protocol and this manuscript, and contributed to the overall trial design. In particular, these co-authors made significant contributions on the design of web-based database and approved final version of the database. N Lee, T-J Cha, T-H Yang, M-H Jeong, M-H Yoon, S U Lee, S J Lee, E-S Shin, J W Kim, J-M Cho, K-R Han and W B Pyun

For peer review only

reviewed the study protocol and proposed helpful ideas and information, and these co-authors are in charge of registering the trial protocol to the review boards of each participating institution. Final manuscript of this paper was reviewed and approved by all authors.

Acknowledgements

Not applicable.

For peer review only

Reference

1. Stefanini GG, Holmes DR, Jr. Drug-eluting coronary-artery stents. *The New England journal of medicine* 2013;**368**(3):254-65.
2. Bangalore S, Kumar S, Fusaro M, et al. Short- and long-term outcomes with drug-eluting and bare-metal coronary stents: a mixed-treatment comparison analysis of 117 762 patient-years of follow-up from randomized trials. *Circulation* 2012;**125**(23):2873-91.
3. Palmerini T, Biondi-Zoccai G, Riva DD, et al. Stent thrombosis with drug-eluting and bare-metal stents: evidence from a comprehensive network meta-analysis. *The Lancet* 2012;**379**(9824):1393-402.
4. Foin N, Lee RD, Torii R, et al. Impact of stent strut design in metallic stents and biodegradable scaffolds. *Int J Cardiol* 2014;**177**(3):800-8.
5. Hamon M, Niculescu R, Deleanu D, et al. Clinical and angiographic experience with a third-generation drug-eluting Orsiro stent in the treatment of single de novo coronary artery lesions (BIOFLOW-I): a prospective, first-in-man study. *EuroIntervention* 2013;**8**(9):1006-11.
6. Hausleiter J, Kastrati A, Wessely R, et al. Prevention of restenosis by a novel drug-eluting stent system with a dose-adjustable, polymer-free, on-site stent coating. *European heart journal* 2005;**26**(15):1475-81.
7. Pilgrim T, Heg D, Roffi M, et al. Ultrathin strut biodegradable polymer sirolimus-eluting stent versus durable polymer everolimus-eluting stent for percutaneous coronary revascularisation (BIOSCIENCE): a randomised, single-blind, non-inferiority trial. *The Lancet* 2014;**384**(9960):2111-22.
8. Massberg S, Byrne RA, Kastrati A, et al. Polymer-free sirolimus- and probucol-eluting versus new generation zotarolimus-eluting stents in coronary artery disease: the Intracoronary Stenting and Angiographic Results: Test Efficacy of Sirolimus- and Probucol-Eluting versus Zotarolimus-eluting Stents (ISAR-TEST 5) trial. *Circulation* 2011;**124**(5):624-32.
9. Windecker S, Kolh P, Alfonso F, et al. 2014 ESC/EACTS Guidelines on myocardial revascularization: The Task Force on Myocardial Revascularization of the European Society of Cardiology (ESC) and the European Association for Cardio-Thoracic Surgery (EACTS) Developed with the special contribution of the European Association of Percutaneous Cardiovascular Interventions (EAPCI). *European heart journal* 2014;**35**(37):2541-619.
10. Mehran R, Rao SV, Bhatt DL, et al. Standardized bleeding definitions for cardiovascular clinical trials: a consensus report from the Bleeding Academic Research Consortium. *Circulation* 2011;**123**(23):2736-47.
11. Hicks KA, Tcheng JE, Bozkurt B, et al. 2014 ACC/AHA Key Data Elements and Definitions for Cardiovascular Endpoint Events in Clinical Trials: A Report of the American College of Cardiology/American Heart Association Task Force on Clinical Data Standards (Writing Committee to Develop Cardiovascular Endpoints Data Standards). *Circulation* 2015;**132**(4):302-61.
12. McAlister. FA, Straus. SE, Sackett. DL, et al. Analysis and reporting of factorial trials: a systematic review. *JAMA* 2003;**289**(19).
13. Windecker S, Haude M, Neumann FJ, et al. Comparison of a novel biodegradable polymer sirolimus-eluting stent with a durable polymer everolimus-eluting stent: results of the randomized BIOFLOW-II trial. *Circulation Cardiovascular interventions* 2015;**8**(2):e001441.
14. Byrne RA, Mehilli J, Iijima R, et al. A polymer-free dual drug-eluting stent in patients

For peer review only

with coronary artery disease: a randomized trial vs. polymer-based drug-eluting stents. *European heart journal* 2009;**30**(8):923-31.

15. Patrick W. Serruys SS, Scot Garg, Robert Jan van Geuns, Gert Richardt, Pawel E. Buszman, Henning Kelbæk, Adrianus Johannes van Boven, Sjoerd H. Hofma, Axel Linke, Volker Klauss, William Wijns,Carlos Macaya, Philippe Garot, Carlo DiMario, Ganesh Manoharan, Ran Kornowski, Thomas Ischinger, Antonio Bartorelli, Jacintha Ronden, Marco Bressers, Pierre Gobbens, Manuela Negoita, Frank van Leeuwen, Stephan Windecker. Comparison of Zotarolimus-Eluting and Everolimus-Eluting Coronary Stents. *The New England journal of medicine* 2010;**363**:136-46.
16. von Birgelen C, Basalus MW, Tandjung K, et al. A randomized controlled trial in second-generation zotarolimus-eluting Resolute stents versus everolimus-eluting Xience V stents in real-world patients: the TWENTE trial. *Journal of the American College of Cardiology* 2012;**59**(15):1350-61.
17. Uchida T, Popma J, Stone GW, et al. The clinical impact of routine angiographic follow-up in randomized trials of drug-eluting stents: a critical assessment of "oculostenotic" reintervention in patients with intermediate lesions. *JACC Cardiovasc Interv* 2010;**3**(4):403-11.
18. Kim BK, Hong MK, Shin DH, et al. A new strategy for discontinuation of dual antiplatelet therapy: the RESET Trial (REal Safety and Efficacy of 3-month dual antiplatelet Therapy following Endeavor zotarolimus-eluting stent implantation). *Journal of the American College of Cardiology* 2012;**60**(15):1340-8.
19. Feres F, Costa RA, Abizaid A, et al. Three vs twelve months of dual antiplatelet therapy after zotarolimus-eluting stents: the OPTIMIZE randomized trial. *JAMA* 2013;**310**(23):2510-22.
20. Levine GN, Bates ER, Blankenship JC, et al. 2011 ACCF/AHA/SCAI Guideline for Percutaneous Coronary Intervention. A report of the American College of Cardiology Foundation/American Heart Association Task Force on Practice Guidelines and the Society for Cardiovascular Angiography and Interventions. *Journal of the American College of Cardiology* 2011;**58**(24):e44-122.
21. Moore P, Barlis P, Spiro J, et al. A randomized optical coherence tomography study of coronary stent strut coverage and luminal protrusion with rapamycin-eluting stents. *JACC Cardiovasc Interv* 2009;**2**(5):437-44.
22. Stefanini GG, Taniwaki M, Windecker S. Coronary stents: novel developments. *Heart* 2014;**100**(13):1051-61.
23. Wessely R, Hausleiter J, Michaelis C, et al. Inhibition of neointima formation by a novel drug-eluting stent system that allows for dose-adjustable, multiple, and on-site stent coating. *Arteriosclerosis, thrombosis, and vascular biology* 2005;**25**(4):748-53.
24. Seung-Jung Park, Duk-Woo Park, Young-Hak Kim, et al. Duration of Dual Antiplatelet Therapy after Implantation of Drug-Eluting Stents. *The New England journal of medicine* 2010;**2010**(362):1374-82.
25. Gwon HC, Hahn JY, Park KW, et al. Six-month versus 12-month dual antiplatelet therapy after implantation of drug-eluting stents: the Efficacy of Xience/Promus Versus Cypher to Reduce Late Loss After Stenting (EXCELLENT) randomized, multicenter study. *Circulation* 2012;**125**(3):505-13.
26. Valgimigli M, Campo G, Monti M, et al. Short- versus long-term duration of dual-antiplatelet therapy after coronary stenting: a randomized multicenter trial. *Circulation* 2012;**125**(16):2015-26.
27. Mauri L, Kereiakes DJ, Yeh RW, et al. Twelve or 30 months of dual antiplatelet therapy after drug-eluting stents. *The New England journal of medicine* 2014;**371**(23):2155-66.

28. Bonaca MP, Bhatt DL, Cohen M, et al. Long-term use of ticagrelor in patients with prior myocardial infarction. *The New England journal of medicine* 2015;**372**(19):1791-800.

For peer review only

Appendix

Endpoint definitions

To gauge the clinical efficacy of each stent system, TLF as a composite of cardiac death, TVMI, and clinically driven TLR will be used as a surrogate for outcome measure. Though 1-year TLF rate will be presented as a primary endpoint of this trial, TLF and its components will be collected up to 3 years to delineate long-term clinical outcome. At the same time, NACE as a comprehensive outcome related to safety issue will be verified throughout the study period and it will disclose the consequences according to the two schemes of mandatory DAPT regimen. Definite or probable stent thrombosis and major bleeding as well as TLF will be regarded as components of NACE. Outcome measures and their components in this trial will follow the current data standards and regulatory guidelines [10 11].

1. Target lesion failure (TLF)

   The composite of clinically driven TLR, MI or cardiac death related to the target vessel. Target lesion is the segment where coronary intervention was performed, and the length of the target lesion is inclusive of the arterial segment treated with the stent and the 5mm proximal and 5mm distal to the treated section. Target vessel is the major native coronary artery or bypass graft containing the target lesion. A native coronary artery target vessel includes the arterial segments upstream and downstream from the target lesion and their major side branches. If it cannot be determined with certainty whether an MI or death is related to the target vessel, and at the same time if no other specific reasons can be given, it will be considered as a case of TLF.

2. Cardiac death

   Death by any cardiovascular mechanisms (arrhythmia, sudden death, low cardiac output heart failure, stroke, pulmonary embolism, or peripheral artery disease) will be

For peer review only

counted as a cardiac death. Unwitnessed death in a subject seen alive and clinically stable less than 24 hours before being found dead without any evidence supporting a specific non-cardiovascular cause of death will be also judged as a cardiac death. In addition, death caused by the immediate complications of the procedure will be managed as a cardiac death. Any death not covered by the above definitions, including death due to natural progression of underlying chronic disease, infection, accident, suicide or trauma will be handled as a non-cardiovascular death.

3. Target vessel myocardial infarction (TVMI)

Among the case of acute spontaneous MI, TVMI is defined as a MI case with the evidence of myocardial necrosis in the vascular territory of previously treated target vessel. As well as direct evidence of invasive angiography, electrocardiographic or other imaging evidences such as echocardiography (e.g., newly developed regional wall motion abnormality or extension of previous abnormality) can be used to adjudicate the involvement of target vessel territory. Any types of MI related to stent thrombosis or restenosis of the target lesion will be included to TVMI case, but periprocedural MI (e.g., type 4a MI associated with and occurring within 48 hours of coronary intervention) and death with symptoms suggestive of myocardial ischemia but without direct evidence of target vessel involvement will be excluded from the outcome measure of TVMI.

4. Clinically driven target lesion revascularization (TLR)

TLR indicates a revascularization procedure with repeated stenting, balloon angioplasty or surgical bypass grafting for restenosed or occluded culprit target lesion. TLR is clinically driven if the target lesion diameter stenosis is more than 50% by quantitative coronary angiography (QCA) and the subject has clinical or functional

For peer review only

ischemia that cannot be explained by another native coronary or bypass graft lesion. Even in the absence of ischemic symptoms or signs, TLR for a stented lesion with more than 70% diameter stenosis may be also considered clinically driven. And in case of the absence of QCA data or if a stenosis ≤50% is present, TLR may be considered clinically driven if severe ischemic signs and symptoms attributable to the target lesions are present. Meanwhile, repeated intervention or surgical bypass for any segment of a coronary artery containing a target lesion will be counted as a case of target vessel revascularization (TVR), and of course, TLR can be considered as TVR. In this context, target vessel failure (TVF) is defined as a composite of clinically driven TVR, cardiac death and TVMI, and this ancillary measure also will be counted throughout the study period.

5. Definite or probable stent thrombosis (ST)

Definite ST is defined as occurring when clinical presentation is consistent with acute coronary syndrome, and angiographic or pathologic examination with autopsy confirm stent occlusion or thrombus in the stented segment. Angiographic confirmation is the presence of a thrombus at the time of angiography that originates in or from the stent or in the segment 5mm proximal or distal to the stent. Probable ST is defined as any unexplained death occurring within the first 30 days that cannot be attributed to other causes. And irrespective of the time after the index procedure, any MI that is related to the territory of the implanted stent without angiographic confirmation of ST can be regarded as probable ST in the absence of any other obvious cause. Timing of the detection of ST will follow to the ARC (Academic Research Consortium) grading criteria: 0-24 hours after stent implantation is acute ST, >24 hours to 30 days after stenting is subacute ST, >30 days to 1 year after the

For peer review only

procedure is late ST, and more than 1 year after stent implantation is very late ST.

6. Major bleeding

Type 3 or 5 bleeding according to the BARC (Bleeding Academic Research Consortium) criteria will be counted as a major bleeding event. Overt bleeding with hemoglobin drop more than 3 g/dL, any transfusion, procedure-related cardiac tamponade, bleeding requiring surgical intervention or intravenous vasoactive agents and intracranial or intraocular hemorrhage will be recorded as a major bleeding event. Fatal bleeding and subsequent death without no other explainable cause also can be categorized into this class of bleeding.

Stent platforms for this trial

Orsiro™ stent system	
Stent backbone	PRO-Kinetic Energy® stent system
Stent alloy	Cobalt chromium alloy
Passive coating	PROBIO™ amorphous silicon carbide coating
Active coating	BIOlute™ high molecular weight Poly-L-Lactic Acid (PLLA)
Coating dose of sirolimus	1.4µg/mm^2
Stent strut thickness	Nominal diameter 2.25~3.00mm: 60µm (0.0024”)<br>3.50~4.00mm: 80µm (0.0031”)
Nominal diameter	2.25 / 2.50 / 2.75 / 3.00 / 3.50 / 4.00mm
Nominal length	9 / 13 / 15 / 18 / 22 / 26 / 30 / 35 / 40mm
Lesion entry profile	0.017” (0.43mm)
Lesion crossing profile	0.039” (0.99 mm)
Nominal inflation pressure	8 atm
Rate burst pressure	16 atm

Coroflex ISAR™ stent system	
Stent backbone	CX Blue Ultra & Neo® stent system
Stent alloy	Cobalt chromium alloy
Coating dose of sirolimus	1.2µg/mm^2
Stent strut thickness	Nominal diameter 2.00~2.50mm (): 50µm (0.0020”)<br>2.75~4.00mm (CX-Blue Neo): 60µm (0.0024”)
Nominal diameter	2.00 / 2.25 / 2.50 / 2.75 / 3.00 / 3.50 / 4.00mm
Nominal length	2.00~2.50mm stent:

For peer review only

	9 / 14 / 16 / 19 / 24 / 27 / 32mm<br>2.75~4.00mm stent:<br>8 / 13 / 16 / 19 / 24 / 27 / 32mm
Lesion entry profile	0.016” (0.41mm)
Lesion crossing profile	0.031~0.037” (0.79~0.93mm)
Nominal inflation pressure	10 atm
Rate burst pressure	18 atm (15 atm in case of 4.00mm-sized stent)

For peer review only

Figure legend for Figure 1.

Study outline and randomization scheme.

Table title for Table 1.

Inclusion and exclusion criteria for the HOST-IDEA trial

For peer review only

Additional files

Additional files 1: SPIRIT Checklist (DOC 122kb)

Additional file 2: SPIRIT Time schedule (DOC 56kb)

Additional file 3: Informed consent form (Korean version, 50kb)

Additional file 4: IRB documentations

4-1: Seoul National University Hospital

4-2: SoonChunHyang University Cheonan Hospital

4-3: Hallym University Kangnam Sacred Heart Hospital

4-4: Korea University Guro Hospital

4-5: Ajou University Hospital

4-6: Chonnam National University Hospital

4-7: Kwangju Christian Hospital

4-8: Inje University Busan Paik Hospital

For peer review only

Figure 1. Study outline and randomization scheme

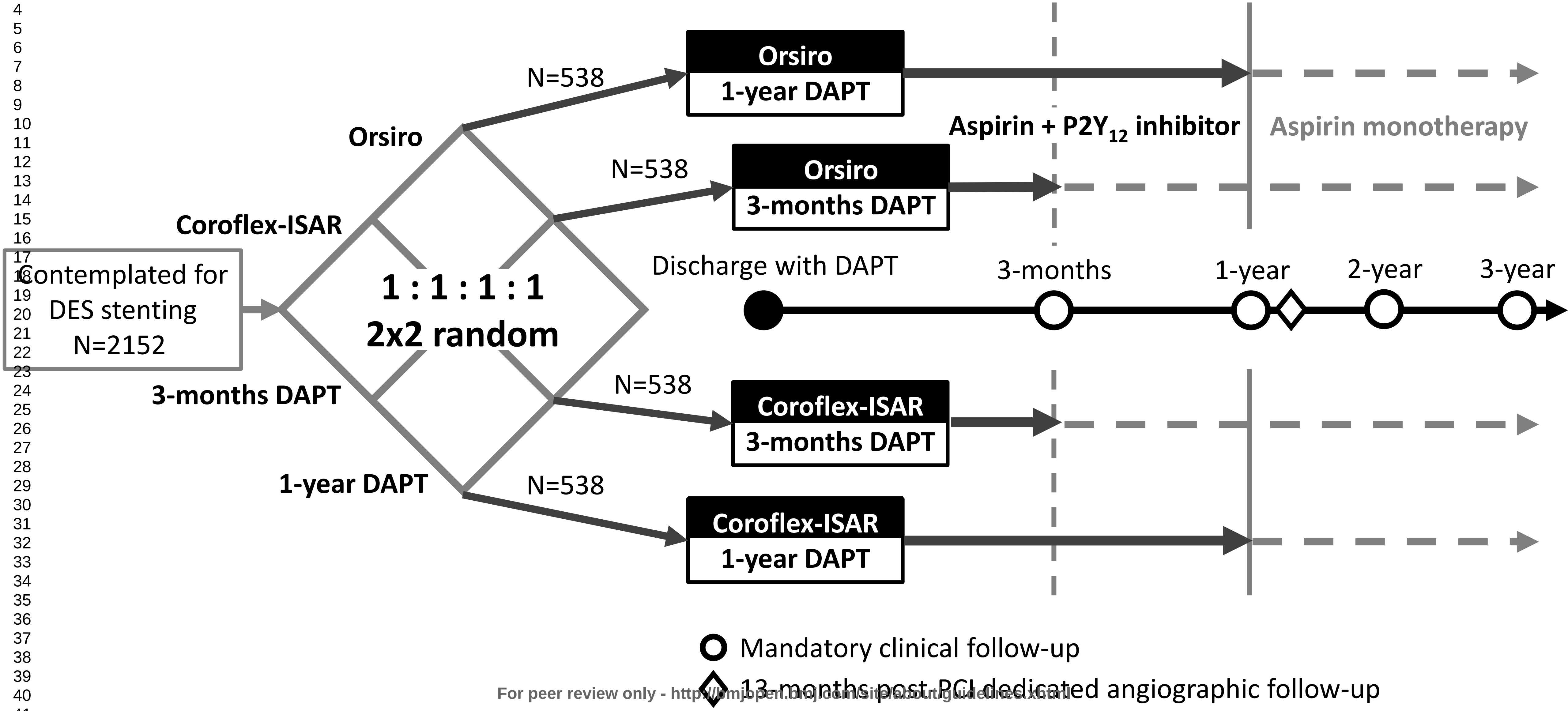

Table 1. Inclusion and exclusion criteria for the HOST-IDEA trial

Inclusion criteria
Patients with de novo stenotic lesions who are suitable for coronary stenting with drug-eluting stent
Exclusion criteria
1. High risk profiles for ischemic adverse events such as<br>A. ST-segment elevation myocardial infarction (STEMI)<br>B. Patients with cardiogenic shock or concomitant severe decompensated heart failure<br>C. Patients with myocardial infarction or stent thrombosis in spite of the maintenance of antiplatelet therapy<br>D. Restenosis in stented segments or previous sites of balloon angioplasty<br>2. Patients who cannot follow allocated DAPT schedule due to the planned surgery or elective procedure within 3 months after the stenting<br>3. Recent history of major surgery or evident events of gastrointestinal bleeding within 1 month from the procedure<br>4. Patients on anticoagulation therapy with warfarin or other anticoagulants<br>5. Life expectancy less than 1 year (such as malignancies or other chronic systemic diseases)<br>6. Pregnant women<br>7. Past history of allergy or other contraindications for the following medications/materials: aspirin, clopidogrel, prasugrel, ticagrelor, heparin, cobalt chromium, sirolimus

For peer review only

Study protocol for a randomized controlled trial: Harmonizing Optimal Strategy for Treatment of coronary artery stenosis – coronary Intervention with next generation Drug-Eluting stent platforms and Abbreviated dual antiplatelet therapy (HOST-IDEA) trial

Journal:	*BMJ Open*
Manuscript ID	bmjopen-2017-016617.R1
Article Type:	Protocol
Date Submitted by the Author:	26-May-2017
Complete List of Authors:	Kim, Chi-Hoon; Seoul National University Hospital<br>Han, Jung-Kyu; Seoul National University Hospital<br>Yang, Han-Mo; Seoul National University Hospital<br>Park, Kyung Woo; Seoul National University Hospital<br>Lee, Hae-Young; Seoul National University Hospital<br>Kang, Hyun-Jae; Seoul National University Hospital<br>Koo, Bon-Kwon; Seoul National University Hospital<br>Lee, Namho; Hallym University Kangnam Sacred Heart Hospital<br>Cha, Tae-Joon; Kosin University Gospel Hospital<br>Yang, Tae-Hyun; Inje University Busan Paik Hospital<br>Jeong, Myung-Ho; Chonnam National University Hospital<br>Yoon, Myeong-Ho; Ajou University Hospital<br>Lee, Seung Uk; Kwangju Christian Hospital<br>Lee, Seung Jin; SoonChunHyang University Cheonan Hospital<br>Kim, Jin Won; Korea University Guro Hospital<br>Cho, Jin-Man; Kyung Hee University Hospital at Gangdong<br>Han, Kyu-Rock; Hallym University Kangdong Sacred Heart Hospital<br>Pyun, Wook Bum; Ewha Womans University Medical Center Mokdong Hospital<br>Kim, Hyo-Soo; Seoul National University Hospital
**Primary Subject Heading**:	Cardiovascular medicine
Secondary Subject Heading:	Cardiovascular medicine
Keywords:	Stent, Polymer, Antiplatelet therapy

SCHOLARONE™
Manuscripts

Study protocol for a randomized controlled trial: Harmonizing Optimal Strategy for Treatment of coronary artery stenosis – coronary Intervention with next generation Drug-Eluting stent platforms and Abbreviated dual antiplatelet therapy (HOST-IDEA) trial

Chi-Hoon Kim,[1*] Jung-Kyu Han,[1*] Han-Mo Yang,[1] Kyung Woo Park,[1] Hae-Young Lee,[1] Hyun-Jae Kang,[1] Bon-Kwon Koo,[1] Namho Lee,[2] Tae-Joon Cha,[3] Tae-Hyun Yang,[4] Myung-Ho Jeong,[5] Myeong-Ho Yoon,[6] Seung Uk Lee,[7] Seung Jin Lee,[8] Jin Won Kim,[9] Jin-Man Cho,[10] Kyu-Rock Han, [11] Wook Bum Pyun,[12] Hyo-Soo Kim[1]

1. Seoul National University Hospital, Seoul, Korea
2. Hallym University Kangnam Sacred Heart Hospital, Seoul, Korea
3. Kosin University Gospel Hospital, Busan, Korea
4. Inje University Busan Paik Hospital, Busan, Korea
5. Chonnam National University Hospital, Gwangju, Korea
6. Ajou University Hospital, Suwon, Korea
7. Kwangju Christian Hospital, Gwangju, Korea
8. SoonChunHyang University Cheonan Hospital, Cheonan, Korea
9. Korea University Guro Hospital, Seoul, Korea
10. Kyung Hee University Hospital at Gangdong, Seoul, Korea
11. Hallym University Kangdong Sacred Heart Hospital, Seoul, Korea

For peer review only

12. Ewha Womans University Medical Center Mokdong Hospital, Seoul, Korea

Correspondence to:

Hyo-Soo Kim, MD, PhD

Phone: 82-2-2072-2226; Fax: 82-2-766-8904; E-mail: hyosoo@snu.ac.kr

* These two authors equally contributed to this work.

* E-mail addresses of co-authors

Chi-Hoon Kim: lightcross@gmail.com

Jung-Kyu Han: hpcrates@gmail.com

Han-Mo Yang: hanname@gmail.com

Kyung Woo Park: kwparkmd@snu.ac.kr

Hae-Young Lee: hylee612@gmail.com

Hyun-Jae Kang: nowkang@snu.ac.kr

Bon-Kwon Koo: bkkoo@snu.ac.kr

Namho Lee: namholee@hallym.or.kr

Tae-Joon Cha: chatjn@gmail.com

For peer review only

Tae-Hyun Yang: yangthmd@naver.com

Myung-Ho Jeong: mhjeong@chonnam.ac.kr

Myeong-Ho Yoon: yoonmh65@hanmail.net

Seung Uk Lee: cardiosu@hanmail.net

Seung Jin Lee: drlsj3@gmail.com

Jin Won Kim: kjwmm@korea.ac.kr

Jin-Man Cho: aceri@medimail.co.kr

Kyu-Rock Han: krheart@hallym.or.kr

Wook Bum Pyun: pwb423@ewha.ac.kr

Hyo-Soo Kim: hyosoo@snu.ac.kr

For peer review only

Abstract

Introduction

We have recently seen the introduction of newer-generation drug-eluting stents (DESs) with ultrathin struts that utilize advanced polymer technologies. However, the efficacy and safety of these newest stents have not yet been fully explored. In addition, there are still controversies over the optimal duration of dual antiplatelet therapy (DAPT) after stent implantation, particularly for ultrathin stents with the newest polymer technologies.

Methods and analysis

The HOST-IDEA trial is a randomized, open-label, multicenter, non-inferiority trial, and the first study to directly compare 2 of these ultrathin sirolimus-eluting stents (SES): Orsiro stent with biodegradable polymer, and polymer-free Coroflex ISAR (CX-ISAR) stent. This study has a scheme of 2×2 factorial design according to the stent type and DAPT duration (3- vs. 12-months). A total of 2,152 patients will be randomized and stratified to demonstrate the non-inferiority of CX-ISAR to Orsiro, or of the abbreviated DAPT duration to the conventional 12 months (both in 1:1 ratio). For the comparison of stent type, primary endpoint is target lesion failure (TLF) which is a composite of cardiac death, target vessel-related myocardial infarction, and clinically driven target lesion revascularization. For the comparison of DAPT duration, net adverse clinical event is the co-primary endpoint which is defined as a composite of TLF, definite/probable stent thrombosis and major bleeding.

Ethic approval and dissemination

All the institutions involved in this study are required to have ethical approval prior to patient enrollment. This multicenter study will recruit patients through competitive registration, but institutions that have not yet obtained ethical approvals have made it impossible to enroll

For peer review only

patients in a centralized Web database. The final results will be presented at relevant international conferences and will be materialized in the form of papers.

Trial registration number

NCT02601157; Pre-results.

Keywords

Stent, Polymer, Antiplatelet therapy

For peer review only

Strengths and limitations of this study

1. To the best of our knowledge, the clinical outcome of two up-to-date coronary stents with ultrathin strut, the Orsiro and Coroflex ISAR stents will be firstly compared in this randomized clinical trial. These two stents are based on the latest drug coating technology, however, no randomized studies have been reported to directly compare these two stents.
2. We could derive a meaningful result on the optimal duration of dual antiplatelet therapy (DAPT) in stents using ultrathin strut by adopting a 2x2 factorial design with a difference in the duration of DAPT maintenance (3- vs. 12-month). This study will confirm that the clinical performance is not worse than the conventional 12-month maintenance even if the DAPT maintenance period is kept short in the latest stents with thin strut thickness.
3. We also will be able to simultaneously test the difference between two co-primary outcomes, target lesion failure and net adverse clinical outcome, since we will register sufficient numbers of patients to secure statistical power.
4. To minimize potential risks and ensure patient safety, patients with ST-segment myocardial infarction (STEMI) who are generally recommended to apply a DAPT maintenance period of one year or more will be excluded from this study.

For peer review only

Introduction

Second generation drug-eluting stents (DESs) has been introduced to overcome the limitations of earlier versions of DESs such as late stent thrombosis and late catch-up.[1] The improvements of second generation DESs were made in many different fields as follows: better stent design with greater conformability, thinner strut thickness by using of new metal alloy, optimal load and improved release kinetics of drugs, and new polymer technology. All these improvements made second generation DESs safer and more efficacious.[2 3]

Thin strut thickness makes greater conformability, better deliverability, and lesser injury. This creates lesser shear disturbances, and reduces peri-strut inflammation and fibrin deposition, finally contributing to improved re-endothelialization.[4] Currently, the Orsiro hybrid sirolimus-eluting stent (SES) (Orsiro, Biotronik AG, Bülach, Switzerland) and the Coroflex ISAR (CX-ISAR, B. Braun Melsungen AG, Berlin, Germany) SES are two commercially available stents with thinnest strut thickness (60μm for diameter ≤3.0mm, 80 μm for diameter ≥3.5mm for Orsiro, 50μm for diameter ≤2.5mm, 60 μm for diameter ≥2.75mm for CX-ISAR). Interestingly, these two stent systems adopt two different up-to-date drug coating technologies. The Orsiro utilizes poly-L-lactic acid (PLLA) for biodegradable polymer.[5] While, sirolimus of the ISAR platform is coated on the stent strut without any polymer substance.[6]

The Orsiro stent showed non-inferior safety and efficacy outcomes compared with everolimus-eluting stents (EESs).[7] In contrast, previous version of the ISAR stent with stainless steel backbone was compared with zotarolimus-eluting stent (ZES), demonstrating its non-inferior safety and efficacy.[8] However, CX-ISAR, a latest version of the ISAR system with cobalt chromium (CoCr) alloy backbone, has not yet been tested in a large scale randomized controlled trial. Furthermore, up to now, there is no head-to-head comparison

For peer review only

between these two ultrathin stents with distinct drug eluting technologies, the Orsiro and CX-ISAR.

Meanwhile, optimal duration of dual antiplatelet therapy (DAPT) after DES implantation is still unconcluded. Current guidelines recommend 6-12 months of DAPT after DES implantation for patients with stable angina.[9] However, there are controversial studies regarding longer- versus shorter-duration DAPT. In particular, we have no data regarding optimal duration of DAPT for ultrathin stents with advanced polymer technologies. Because of potential of better re-endothelialization and less polymer-related adverse effects,[1 4] we can reasonably guess that shortened DAPT would be enough for these newer generation stents.

Therefore, this prospective, randomized, open-label, 2×2 factorial design multicenter trial was planned to address: (1) whether newly-developed ultrathin stents (Orsiro, CX-ISAR) are comparable to each other in terms of efficacy and safety, and (2) whether short duration of DAPT is non-inferior to conventional 1 year duration in patients receiving ultrathin newer generation DESs.

For peer review only

Methods and design

Study design and primary hypothesis

The HOST-IDEA trial is a randomized, open-label, multicenter, non-inferiority trial comparing the Orsiro with the CX-ISAR. The trial is powered to investigate a hypothesis that a polymer-free stent platform (CX-ISAR) is non-inferior to a biodegradable polymer-based stent (Orsiro) as regards post-procedure 1-year target lesion failure (TLF), as a composite of cardiac death, target vessel myocardial infarction (TVMI), and clinically driven target lesion revascularization (TLR). At the same time, another hypothesis will also be examined: 3 months' DAPT may deliver the same clinical efficacy and safety as conventional 1-year DAPT strategy. For this purpose, net adverse clinical events (NACE), defined as a composite of TLF, definite or probable stent thrombosis, and major bleeding according to the pertinent criteria,[10 11] will be checked as a co-primary endpoint. A 2×2 factorial design will be used to address these questions. Unless there is significant interaction between the two interventions, this factorial design can provide a useful scheme for testing two interventions simultaneously in a single dataset, and can be used to minimize sample size without limiting the statistical power.[12]

Study population and eligibility criteria

All participating centers are tertiary referral hospitals in Korea. Patients eligible for coronary intervention will be qualified with coronary angiography before the enrollment. Every participant will have at least 1 stenotic coronary lesion of >50% diameter stenosis suitable for stent implantation. To secure the statistical power and to obtain clear practical implications, high-risk patients for ischemic adverse events will be excluded in this protocol; patients with

For peer review only

ST-segment elevation myocardial infarction (STEMI) or unstable conditions such as cardiogenic shock or severe heart failure at the time of presentation. Detailed criteria for inclusion and exclusion are listed in Table 1.

Rationale for sample size calculation

Among the contemporary DESs, no stent platform has been directly compared to both Orsiro and CX-ISAR. And, the latest version of the ISAR system with a CoCr backbone, CX-ISAR, has not yet been tested in a large-scale randomized controlled trial (RCT). Instead, the Orsiro and previous versions of the ISAR system have been tested against EES and ZES, respectively, and clinical outcomes were found to be comparable in a number of large-scale studies.[7 8 13 14] The Orsiro was non-inferior to the Xience EES in the BIOSCIENCE trial,[7] and previous stainless steel-based ISAR platform and the Resolute ZES system had similar efficacy in the ISAR-TEST 5 trial.[8] As ZES and EES have similar efficacy in treating coronary disease,[15 16] it is reasonable to assume that the Orsiro and ISAR system will have similar levels of clinical efficacy and safety.

Other evidence supports this assumption. The TLF rate of Orsiro in the BIOSCIENCE trial was 6.5% over a 1-year follow-up.[7] In contrast, the ISAR system had a 1-year TLF rate of 13.1% in the ISAR-TEST 5 study.[8] This discrepancy was mainly due to the difference in TLR, rather than cardiac death or TVMI. The TLR rate of the ISAR system was somewhat higher than that of the Orsiro (1.9% cardiac deaths in the Orsiro vs. 1.9% in the ISAR system, TVMI 2.9% vs. 2.4%, TLR 4.0% vs. 10.3%, respectively). However, this contrast may be due to the study's policy regarding angiographic follow-up rather than the nature of the stent system itself. In the ISAR-TEST 5 trial, 6–8 months after the procedure, about three-quarters of patients (76.3%) had undergone dedicated angiographic surveillance,[8] whereas in the

For peer review only

BIOSCIENCE trial, angiography was performed at the time of 13-month follow-up. Consequently, 1-year clinical outcomes of the Orsiro could avoid the potential influence of routine angiographic follow-up, and this trial was able to minimize the risk of oculostenotic revascularization for non-ischemic intermediate lesions.[17] In a similar vein, 1-year TLR rate of 10.4% for the Resolute ZES system was also reported in the ISAR-TEST 5 trial, but in the Resolute all-comer study, without the impact of routine angiographic follow-up within 12 months, the same stent system had a lower TLR rate of 3.9%.[15]

Based on these data, it is reasonable to assume that the CX-ISAR and Orsiro would have similar TLR rates. Accordingly, 1-year TLF rate of the Orsiro in the BIOSCIENCE trial was employed as a reference for power calculation. With the assumption of a TLF rate of 6.5% and allowing for about 10% withdrawals or dropouts, a total of 2152 patients in 1:1 randomization will provide more than 80% power to detect a non-inferiority margin of 2.8% with a one-sided type I error of 0.05. These parameters are comparable to those of the BIOSCIENCE trial (reference value and non-inferiority margin 8.0% and 3.5%, respectively).[7] This size calculation may be able to secure the statistical power in case the event rates may be lower than expected. Non-inferiority margin of 2.5% for event rate of 5%, or non-inferior margin of 2.7% for event rate of 6% can be examined with this sample size even allowing for 10% withdrawal or dropouts.

This sample size might also be sufficient to test the second hypothesis. To date, no detailed data on the NACE rate of the CX-ISAR system have been reported. For the Orsiro stent, because most cases of stent thrombosis can also be counted as TLF events (cardiac death or MI), rates of 6.5% for TLF and 3.0% for major bleeding could be cited as references for 1-year NACE rate. Assuming patients with the Orsiro and 1-year DAPT have a NACE rate of 9.5%, 1039 patients will be required for each group of 3-months vs. 1-year DAPT to

For peer review only

distinguish a 3.2% margin of non-inferiority with an α value of 0.05 and 80% power. There is no actual interaction between the two interventions of randomization, maintenance duration of DAPT and allocated stent types. Therefore, even though the sample size of 2152 patients might be insufficient to tell the difference of individual components of NACE, this sample size might cover some patient loss and could provide enough statistical power to verify the second hypothesis simultaneously in a 2×2 factorial design. The 3.2% non-inferiority margin is similar to the reference values of the RESET and OPTIMIZE trials.[18 19]

Enrollment, procedure and study medications

After the identification of the target lesion and patients' consent to participate, randomization to stent type and DAPT duration will be performed using an electronic web-based database (© Cardiovascular Center, Seoul National University Hospital, and CRSCube Software, Seoul, South Korea) according to the 2×2 factorial design (Figure 1). Block randomization with block size of 8 and equal allocation probability for each group will be maintained throughout the entire study period. To ensure the randomization more secure, we have set a small block size for this study, and have not stratified by each participating center. All the information about demographic, procedural, and follow-up data will be integrated into this centralized encrypted electronic database. Though this trial is an open-label study, these data will be managed by independent research nurses or other well-trained professionals.

Coronary intervention will be performed according to generally accepted current guidelines.[9 20] To improve applicability of this trial, DAPT regimen with prasugrel or ticagrelor as well as clopidogrel will be allowed. Every antiplatelet-naïve patient undergoing an elective procedure will be given 300 mg aspirin and loading dose of one of $P2Y_{12}$ receptor inhibitors (e.g., 600 mg clopidogrel, 60 mg prasugrel or 180 mg ticagrelor) preferably ≥2

For peer review only

hours before the intervention. These loading doses can be waived for chronic antiplatelet users. Choice for $P2Y_{12}$ inhibitors will be based on the current guidelines as well as patient/lesional characteristics. To avoid possible bias, clopidogrel will be preferentially used for patients with stable angina whereas prasugrel or ticagrelor will be used mainly for patients with acute coronary syndrome.[9] Patients with higher bleeding risk such as patients older than 75 years of age, history of ischemic stroke or propensity to bleed can be treated with clopidogrel even in the case of acute coronary syndrome.

During the procedure, unfractionated heparin in a dose of at least 5000 IU or 70–100 IU/kg body weight will be administered for anticoagulation, while bail-out glycoprotein IIb/IIIa inhibitors, such as abciximab, will be left to the operators' discretion. Any lesional characteristics will be allowed for enrollment, except for in-stent restenosis of stented segments or previous treatment with balloon angioplasty. In addition to the angiographic findings, additional evaluations, such as intravascular ultrasound, optical coherence tomography (OCT), or fractional flow reserve assessed with pressure wire, may be used to characterize target lesions. Staged intervention can also be reserved for patients with complex lesional characteristics or multivessel disease, but even in these cases, target lesions can be treated only with the allocated stent platform. Because the Orsiro and CX-ISAR systems have similar configuration (see appendix), they are expected to be interchangeable, with no substantial differences in the procedure.

Follow-up and data collection

A DAPT schedule according to the web-based randomization is mandatory for every participant. Daily maintenance dose of aspirin is 100 mg and all patients will be given maintenance dose of $P2Y_{12}$ inhibitors according to their allocation (clopidogrel at a dose of

For peer review only

75 mg daily, prasugrel 10mg daily (5mg daily for patients with body weight of less than 60 kg) or ticagrelor 90mg twice a day). DAPT will be continued for up to 3 months or 1 year, as planned. To check patient's adherence to the DAPT regimen, a drug compliance survey will be conducted at the 1- and 3-month clinical follow-ups. Within this initial 3 months of follow-up, any unexpected discontinuation of DAPT will be regarded as a violation of the study protocol. After 3-month follow-up, brief interruptions of ≤5 days for elective surgery or planned procedures will be permitted, but every interruption of ≥6 days will be classified as non-adherence to the allocated 1-year DAPT regimen. This issue will be addressed for every case during the follow-up. Clinical follow-ups at the time of 1-, 3- and 12-month after the study enrollment are mandatory for the completion of the study. And during the period between 3 and 12 months, every patient will be required for out-patient visits at an interval less than 3 months. In addition to survey for DAPT compliance at the time of every out-patient visit, telephone interview will be given to patients who miss their scheduled appointments. DAPT with aspirin and clopidogrel/prasugrel/ticagrelor may be also extended beyond the predefined period, according to the patient's risk and the responsible clinician's discretion. Allocated $P2Y_{12}$ inhibitors will not be changed to other agents during the entire study period. But for patients with higher ischemic risk, prasugrel or ticagrelor may replace clopidogrel after the predefined period of DAPT maintenance.

Regardless of violation or drop-out, clinical outcomes as well as drug compliance up to 3 years will be collected in the centralized web database; data entry will be assigned to independent professionals. Any serious adverse events, including death, MI, revascularization, stent thrombosis, and bleeding will be entered into the web database for up to 3 years in a blinded fashion. These events will be adjudicated independently by a blinded adjudication committee. Central and on-site data monitoring will be performed according to a predefined monitoring plan. Every electronic case report form will be checked by central data

For peer review only

monitoring. On-site monitoring will also be performed to secure data integrity; records of the first 10 patients and subsequently a random 20% of the total registered patients will be verified. Dedicated angiographic surveillance will be scheduled at the time of 13-month follow-up, but this is not mandatory. Detailed instructions will be provided to each institution.

Statistical analysis

Interim analyses will be performed to test the feasibility of this trial when half of the enrolled patients have their 1-year results. Potential interactions between the stent types and the recommended maintenance duration of DAPT will be identified to ensure the statistical power of this trial, before the study hypotheses are addressed.[12] The primary outcome will be examined from an intention-to-treat viewpoint, but considering the potential influences of protocol violation or drop-out, per-protocol analysis will be used at the same time. The per-protocol population will be limited to patients with (1) a successful procedure treated solely with the allocated stent type, (2) no violation of recommended antiplatelet strategy, and (3) complete clinical follow-up information.

Using the proportional-hazards model, clinical outcomes will be compared between the stent types and DAPT strategies, possibly after controlling for relevant covariates. Stratified analyses for the primary endpoint across major subgroups will be performed using the Mantel–Cox method. Subgroup analyses will be stratified by male sex, smoking history, diabetes mellitus, chronic kidney disease of stage ≥3, off- versus on-label indication, small vessel (≤2.75 mm) or long target lesion (>28 mm), and complex lesion (type B2/C) or chronic total obstruction. Rates of bleeding complications will be analyzed according to the allocated antiplatelet strategy.

For peer review only

Definitions of outcome

Outcome measures and endpoint concepts will follow the definitions suggested in current recommendations.[11] The primary endpoint in this trial is TLF, as defined above, while the composite outcome of NACE will be managed as a co-primary endpoint. Detailed definitions are summarized in the appendix.

For peer review only

Discussion

The HOST-IDEA RCT is the first study to directly compare 2 ultrathin CoCr backbone stents with up-to-date polymer technologies: the sirolimus-eluting Orsiro stent with biodegradable polymer, and the sirolimus-eluting polymer-free CX-ISAR stent. These two stents are clearly distinct from other contemporary drug-eluting stents (DES) with durable polymers. Strut thickness is 50-60μm for Orsiro stent, and 60-80μm for CX-ISAR stent. Ultrathin strut thickness makes these stents more conformable and deliverable. In addition, injuries to the arterial wall during stent implantation and subsequent peri-strut inflammation can be minimized.[4] This feature may also contribute to more rapid arterial healing and more reliable endothelial coverage.[13 21] Further, these two stent platforms have adopted different up-to-date polymer technologies. Orsiro stent utilizes two-tiered hybrid polymer coating technology.[13 22] The outer layer is made of poly-L-lactic acid, which completely degrades over about 1 year period. The inner layer is silicon carbide inert matrix, which prevents the CoCr struts from being exposed to the diseased segment.[5] This unique hybrid system may greatly reduce chronic local inflammation around the stent struts and lessen the risk of denuded struts without re-endothelialization. In contrast, CX-ISAR utilizes polymer-free drug release technology. A mixture of sirolimus, probucol, and shellac resin is mounted into numerous micropores on the stent strut.[6 23] Since the sirolimus in this dual drug-delivery system has the same eluting profile to that of the lipophilic solvent probucol, controlled drug release is enabled for up to 6-8 weeks, and nothing will be left on the stent struts after 3 months.

Few previous studies assessed the clinical outcome of the Orsiro stent. The BIOSCIENCE trial randomized 1,063 patients to Orsiro and 1,056 patients to Xience EES stent.[7] Clinical efficacy of the Orsiro stent was comparable to that of EES, widely used durable polymer stent (1-year TLF rate in Orsiro 6.5% vs. 6.6% in EES group, *P* for non-inferiority <0.0004). The

safety profile of the Orsiro was also reliable: only 9 cases (0.9%) were reported as definite stent thrombosis during the 1-year follow-up period, compared to 4 cases (0.4%) in EES group (P = 0.16). Interestingly, in this trial, STEMI patients treated with the Orsiro were associated with a lower risk for 1-year TLF: 7 cases (3.3%) of 211 STEMI patients in Orsiro group vs. 17 (8.7%) of 196 in EES group (rate ratio 0.38, 95% CI 0.16–0.91, P for interaction 0.014). In contrast, the efficacy and safety of CX-ISAR have not been examined yet in a large-scale RCT. The HOST-IDEA RCT will provide the data regarding the efficacy and safety of the Orsiro and CX-ISAR by comparing these two stents.

In addition, there is still controversy over the optimal duration of DAPT following DES implantation. Several previous trials demonstrated that a shortened DAPT strategy was comparable to conventional 1 year strategy. The REAL-LATE/ZEST-LATE trials analyzed 2,701 patients who had received SES (57%), paclitaxel-eluting stent (PES) (24%), ZES (19%) or other DESs.[24] The results demonstrated that DAPT longer than 12 months was not more effective compared with aspirin monotherapy to reduce the rate of myocardial infarction (MI) or cardiac death. Our group previously compared 6-month to 12-month DAPT in 1,443 patients who underwent Xience/Promus EES or Cypher SES implantation in the EXCELLENT RCT.[25] We revealed that 6-month DAPT did not increase the risk of target vessel failure or the incidence of safety endpoint. And the RESET trial showed that 3-month DAPT following Endeavor ZES implantation was non-inferior to 12-month DAPT following other DES in 2,117 patients with respect to the occurrence of the primary endpoint consisting of cardiovascular death, MI, stent thrombosis, target/vessel revascularization, or bleeding.[18] In the PRODIGY trial, 2,013 patients who received bare-metal stent, PES or EES were randomly allocated to take 6-month or 24-month DAPT. As a result, a 24-month DAPT was not more effective than 6-month regimen in reducing the composite of all-cause mortality, MI or cerebrovascular accident, whereas there was a greater risk of bleeding in the 24-month

For peer review only - http://bmjopen.bmj.com/site/about/guidelines.xhtml

group.[26] In the OPTIMIZE trial including 3,119 patients after ZES implantation, 3-month DAPT was non-inferior to 12-month therapy for the primary endpoint composed of all-cause mortality, MI, stroke, or major bleeding.[19] In contrast, some recent trials demonstrated that prolonged DAPT significantly reduced thrombotic adverse clinical events. In the DAPT trial, 9,961 patients after 12-month DAPT following DES implantation were randomly assigned to continue DAPT or not. DAPT beyond 1 year after DES placement reduced the risks of stent thrombosis and major adverse cardiovascular and cerebrovascular events at the expense of an increased risk of bleeding.[27] The PEGASUS-TIMI 54 trial randomly allocated 21,162 patients with MI more than 1 year earlier to take an additional ticagrelor or placebo on top of aspirin. The results showed that prolonged DAPT using ticagrelor reduced the risk of cardiovascular death, MI, or stroke, but increased rates of major bleeding.[28]

Regarding this DAPT issue, the HOST-IDEA RCT also examines the safety and efficacy of the abbreviated DAPT duration in DAPT duration arm. Particularly, this trial will provide the first evidence regarding the optimal DAPT duration for the ultrathin stents with the newest polymer technologies. In addition, this study may also provide meaningful data on the clinical usefulness of the 3-month DAPT regimen for small-vessel intervention. In fact, the intervention for small-vessel diameter (<3mm) is an item of the DAPT score and is included in the model to predict the occurrence of the future ischemic adverse events.[29] In this regard, the recent guideline has advised that long-term DAPT maintenance should be considered for small-vessel intervention.[30] But, there is data that contradict this recommendation. Some previous studies such as the RESET and OPTIMIZE trials have shown that the 3-month DAPT regimen is clinically useful and safe even in small-vessel intervention.[18 19] However, these studies used the Endeavor and Resolute zotarolimus-eluting stents, which are no longer used in the current clinical practice. To date, detailed data on the effect of the combination of the 3-month DAPT regimen and the new stent platforms on small-vessel intervention are very

For peer review only

scarce. In this context, even if the 3-month DAPT regimen were used for the Orsiro or the CX-ISAR stents, we expect that excellent clinical outcome can also be achieved in small-vessel intervention.

For peer review only

Ethical approval and status of the trial

Ethics approval and consent to participate

At the time of submission (February 2017), a total of 12 centers are participating in this trial. This prospective trial had been approved from 8 centers, as of February 2017, name of the regulation authority and the issued IRB number are as follows: the review board of Seoul National University Hospital (D-1508-118-697), SoonChunHyang University Cheonan Hospital (SCHCA 2016-03-012), Hallym University Kangnam Sacred Heart Hospital (2016-04-31), Korea University Guro Hospital (MD16043), Ajou University Hospital (AJIRB-MED-DE4-16-170), Chonnam National University Hospital (CNUH-2016-096), Kwangju Christian Hospital (KCH-D-2016-03-003), Inje University Busan Paik Hospital (16-0117). Other 4 centers are on the review process with the same protocol and consent form (Kosin University Gospel Hospital, Kyung Hee University Hospital at Gangdong, Ewha Womans University Medical Center Mokdong Hospital, Hallym University Kangdong Sacred Heart Hospital). Recruitment will not begin in any of these 4 centers until all local approvals have been obtained. The steering committee of this trial takes the responsibility for the study design. 12 independent regulation authorities of this trial are in full compliance with Good Clinical Practice as defined under the Korean Ministry of Food and Drug Safety regulations and the International Conference on Harmonization guidelines.

All patients will receive sufficient information to make a decision about participation before providing their written informed consent. Informed consent will be obtained by independent research nurses of well-trained personnel of each participating center. And every participant will have the right to withdraw their consent without restriction. Deferred consent will not be permitted for this study. Consent to publication will be obtained as a part of the general consent form, and individual patient data will be processed anonymously.

For peer review only

Trial registration and current status of this trial

This trial was registered at clinicaltrials.gov (NCT02601157; November 7, 2015). On January 28, 2016, we enrolled patients for the first time at the coordinating center (Seoul National University Hospital), since then 6 participating centers have begun to register patients. A total of 143 patients had been enrolled as of February 2017, and we expect patient registration will be extended to other institutions by the end of this year. This paper translates the study protocol version 1.0. Any protocol amendments or revisions will be communicated with researchers involved in this trial and mentioned in the results paper.

Availability of data and material

This article is distributed under the terms of the Creative Commons Attribution 4.0 International License (http://creativecommons.org/licenses/by/4.0/), which permits unrestricted use, distribution, and reproduction in any medium, provided you give appropriate credit to the original author(s) and the source, provide a link to the Creative Commons license, and indicate if changes were made. The Creative Commons Public Domain Dedication waiver (http://creativecommons.org/publicdomain/zero/1.0/) applies to the data made available in this article, unless otherwise stated. But, study results and conclusion of this trial will be separately covered in the results paper, and the dataset of this trial will not be shared unless otherwise stated.

For peer review only

Funding

This study was supported by the Korea Health Technology R&D Project (the grant from the 'Korea research-driven hospital' project, HI14C1277) through the Korea Health Industry Development Institute (KHIDI), funded by the Ministry of Health & Welfare (MHW). And, this study is an investigator-initiated trial which is partly supported by an unrestricted grant from Biotronik Korea (Seongnam-si, Gyeonggi-do, South Korea) and B.Braun Korea (Seoul, South Korea), . These two companies equally contributed to this study, but funding source did not involve in the study design. And they will have no role in the data collection and management. Data analysis, its interpretation and publication will be done without any interference.

Competing interests

All researchers, including the chair of this study – Hyo-Soo Kim, M.D., PhD., have no conflict of interest.

Authors' contributions

Dr. H-S Kim, as a corresponding author, proposed the original concept and idea for this HOST-IDEA trial and supervised overall process of preparation. C-H Kim and J-K Han made a draft of the study protocol and prepared this manuscript (These two authors equally contributed this paper.) Patient consent form and centralized web-based database were prepared by C-H Kim. H-M Yang, K W Park, H-Y Lee, H-J Kang and B-K Koo critically reviewed the study protocol and this manuscript, and contributed to the overall trial design. In particular, these co-authors made significant contributions on the design of web-based

For peer review only

database and approved final version of the database. N Lee, T-J Cha, T-H Yang, M-H Jeong, M-H Yoon, S U Lee, S J Lee, J W Kim, J-M Cho, K-R Han and W B Pyun reviewed the study protocol and proposed helpful ideas and information, and these co-authors are in charge of registering the trial protocol to the review boards of each participating institution. Final manuscript of this paper was reviewed and approved by all authors.

Acknowledgements

Not applicable.

For peer review only

References

1. Stefanini GG, Holmes DR, Jr. Drug-eluting coronary-artery stents. *The New England journal of medicine* 2013;368(3):254-65. doi: 10.1056/NEJMra1210816

2. Bangalore S, Kumar S, Fusaro M, et al. Short- and long-term outcomes with drug-eluting and bare-metal coronary stents: a mixed-treatment comparison analysis of 117 762 patient-years of follow-up from randomized trials. *Circulation* 2012;125(23):2873-91. doi: 10.1161/CIRCULATIONAHA.112.097014

3. Palmerini T, Biondi-Zoccai G, Della Riva D, et al. Stent thrombosis with drug-eluting and bare-metal stents: evidence from a comprehensive network meta-analysis. *Lancet* 2012;379(9824):1393-402. doi: 10.1016/S0140-6736(12)60324-9

4. Foin N, Lee RD, Torii R, et al. Impact of stent strut design in metallic stents and biodegradable scaffolds. *Int J Cardiol* 2014;177(3):800-8. doi: 10.1016/j.ijcard.2014.09.143

5. Hamon M, Niculescu R, Deleanu D, et al. Clinical and angiographic experience with a third-generation drug-eluting Orsiro stent in the treatment of single de novo coronary artery lesions (BIOFLOW-I): a prospective, first-in-man study. *EuroIntervention* 2013;8(9):1006-11. doi: 10.4244/EIJV8I9A155

6. Hausleiter J, Kastrati A, Wessely R, et al. Prevention of restenosis by a novel drug-eluting stent system with a dose-adjustable, polymer-free, on-site stent coating. *European heart journal* 2005;26(15):1475-81. doi: 10.1093/eurheartj/ehi405

7. Pilgrim T, Heg D, Roffi M, et al. Ultrathin strut biodegradable polymer sirolimus-eluting stent versus durable polymer everolimus-eluting stent for percutaneous coronary revascularisation (BIOSCIENCE): a randomised, single-blind, non-inferiority trial. *Lancet* 2014;384(9960):2111-22. doi: 10.1016/S0140-6736(14)61038-2

For peer review only

8. Massberg S, Byrne RA, Kastrati A, et al. Polymer-free sirolimus- and probucol-eluting versus new generation zotarolimus-eluting stents in coronary artery disease: the Intracoronary Stenting and Angiographic Results: Test Efficacy of Sirolimus- and Probucol-Eluting versus Zotarolimus-eluting Stents (ISAR-TEST 5) trial. *Circulation* 2011;124(5):624-32. doi: 10.1161/CIRCULATIONAHA.111.026732

9. Authors/Task Force m, Windecker S, Kolh P, et al. 2014 ESC/EACTS Guidelines on myocardial revascularization: The Task Force on Myocardial Revascularization of the European Society of Cardiology (ESC) and the European Association for Cardio-Thoracic Surgery (EACTS)Developed with the special contribution of the European Association of Percutaneous Cardiovascular Interventions (EAPCI). *European heart journal* 2014;35(37):2541-619. doi: 10.1093/eurheartj/ehu278

10. Mehran R, Rao SV, Bhatt DL, et al. Standardized bleeding definitions for cardiovascular clinical trials: a consensus report from the Bleeding Academic Research Consortium. *Circulation* 2011;123(23):2736-47. doi: 10.1161/CIRCULATIONAHA.110.009449

11. Hicks KA, Tcheng JE, Bozkurt B, et al. 2014 ACC/AHA Key Data Elements and Definitions for Cardiovascular Endpoint Events in Clinical Trials: A Report of the American College of Cardiology/American Heart Association Task Force on Clinical Data Standards (Writing Committee to Develop Cardiovascular Endpoints Data Standards). *Circulation* 2015;132(4):302-61. doi: 10.1161/CIR.0000000000000156

12. McAlister FA, Straus SE, Sackett DL, et al. Analysis and reporting of factorial trials: a systematic review. *JAMA* 2003;289(19):2545-53. doi: 10.1001/jama.289.19.2545

13. Windecker S, Haude M, Neumann FJ, et al. Comparison of a novel biodegradable polymer sirolimus-eluting stent with a durable polymer everolimus-eluting stent: results of

For peer review only

the randomized BIOFLOW-II trial. *Circulation Cardiovascular interventions* 2015;8(2):e001441. doi: 10.1161/CIRCINTERVENTIONS.114.001441

14. Byrne RA, Mehilli J, Iijima R, et al. A polymer-free dual drug-eluting stent in patients with coronary artery disease: a randomized trial vs. polymer-based drug-eluting stents. *European heart journal* 2009;30(8):923-31. doi: 10.1093/eurheartj/ehp044

15. Patrick W. Serruys SS, Scot Garg, Robert Jan van Geuns, Gert Richardt, Pawel E. Buszman, Henning Kelbæk, Adrianus Johannes van Boven, Sjoerd H. Hofma, Axel Linke, Volker Klauss, William Wijns,Carlos Macaya, Philippe Garot, Carlo DiMario, Ganesh Manoharan, Ran Kornowski, Thomas Ischinger, Antonio Bartorelli, Jacintha Ronden, Marco Bressers, Pierre Gobbens, Manuela Negoita, Frank van Leeuwen, Stephan Windecker. Comparison of Zotarolimus-Eluting and Everolimus-Eluting Coronary Stents. *The New England journal of medicine* 2010;363:136-46.

16. von Birgelen C, Basalus MW, Tandjung K, et al. A randomized controlled trial in second-generation zotarolimus-eluting Resolute stents versus everolimus-eluting Xience V stents in real-world patients: the TWENTE trial. *Journal of the American College of Cardiology* 2012;59(15):1350-61. doi: 10.1016/j.jacc.2012.01.008

17. Uchida T, Popma J, Stone GW, et al. The clinical impact of routine angiographic follow-up in randomized trials of drug-eluting stents: a critical assessment of "oculostenotic" reintervention in patients with intermediate lesions. *JACC Cardiovasc Interv* 2010;3(4):403-11. doi: 10.1016/j.jcin.2010.01.010

18. Kim BK, Hong MK, Shin DH, et al. A new strategy for discontinuation of dual antiplatelet therapy: the RESET Trial (REal Safety and Efficacy of 3-month dual antiplatelet Therapy following Endeavor zotarolimus-eluting stent implantation). *Journal of the*

For peer review only

American College of Cardiology 2012;60(15):1340-8. doi: 10.1016/j.jacc.2012.06.043

19. Feres F, Costa RA, Abizaid A, et al. Three vs twelve months of dual antiplatelet therapy after zotarolimus-eluting stents: the OPTIMIZE randomized trial. *JAMA* 2013;310(23):2510-22. doi: 10.1001/jama.2013.282183

20. Levine GN, Bates ER, Blankenship JC, et al. 2011 ACCF/AHA/SCAI Guideline for Percutaneous Coronary Intervention. A report of the American College of Cardiology Foundation/American Heart Association Task Force on Practice Guidelines and the Society for Cardiovascular Angiography and Interventions. *J Am Coll Cardiol* 2011;58(24):e44-122. doi: 10.1016/j.jacc.2011.08.007

21. Moore P, Barlis P, Spiro J, et al. A randomized optical coherence tomography study of coronary stent strut coverage and luminal protrusion with rapamycin-eluting stents. *JACC Cardiovasc Interv* 2009;2(5):437-44. doi: 10.1016/j.jcin.2009.01.010

22. Stefanini GG, Taniwaki M, Windecker S. Coronary stents: novel developments. *Heart* 2014;100(13):1051-61. doi: 10.1136/heartjnl-2012-303522

23. Wessely R, Hausleiter J, Michaelis C, et al. Inhibition of neointima formation by a novel drug-eluting stent system that allows for dose-adjustable, multiple, and on-site stent coating. *Arteriosclerosis, thrombosis, and vascular biology* 2005;25(4):748-53. doi: 10.1161/01.ATV.0000157579.52566.ee

24. Seung-Jung Park, Duk-Woo Park, Young-Hak Kim, et al. Duration of Dual Antiplatelet Therapy after Implantation of Drug-Eluting Stents. *The New England journal of medicine* 2010;2010(362):1374-82.

25. Gwon HC, Hahn JY, Park KW, et al. Six-month versus 12-month dual antiplatelet therapy after implantation of drug-eluting stents: the Efficacy of Xience/Promus Versus Cypher to

For peer review only

Reduce Late Loss After Stenting (EXCELLENT) randomized, multicenter study. *Circulation* 2012;125(3):505-13. doi: 10.1161/CIRCULATIONAHA.111.059022

26. Valgimigli M, Campo G, Monti M, et al. Short- versus long-term duration of dual-antiplatelet therapy after coronary stenting: a randomized multicenter trial. *Circulation* 2012;125(16):2015-26. doi: 10.1161/CIRCULATIONAHA.111.071589

27. Mauri L, Kereiakes DJ, Yeh RW, et al. Twelve or 30 months of dual antiplatelet therapy after drug-eluting stents. *The New England journal of medicine* 2014;371(23):2155-66. doi: 10.1056/NEJMoa1409312

28. Bonaca MP, Bhatt DL, Cohen M, et al. Long-term use of ticagrelor in patients with prior myocardial infarction. *The New England journal of medicine* 2015;372(19):1791-800. doi: 10.1056/NEJMoa1500857

29. Yeh RW, Secemsky EA, Kereiakes DJ, et al. Development and Validation of a Prediction Rule for Benefit and Harm of Dual Antiplatelet Therapy Beyond 1 Year After Percutaneous Coronary Intervention. *JAMA* 2016;315(16):1735-49. doi: 10.1001/jama.2016.3775

30. Bittl JA, Baber U, Bradley SM, et al. Duration of Dual Antiplatelet Therapy: A Systematic Review for the 2016 ACC/AHA Guideline Focused Update on Duration of Dual Antiplatelet Therapy in Patients With Coronary Artery Disease: A Report of the American College of Cardiology/American Heart Association Task Force on Clinical Practice Guidelines. *Journal of the American College of Cardiology* 2016;68(10):1116-39. doi: 10.1016/j.jacc.2016.03.512

For peer review only

Figure legend for Figure 1.

Study outline and randomization scheme

For peer review only

Table 1. Inclusion and exclusion criteria for the HOST-IDEA trial

Inclusion criteria
Patients with de novo stenotic lesions who are suitable for coronary stenting with drug-eluting stent
**Exclusion criteria**
1. High risk profiles for ischemic adverse events such as
A. ST-segment elevation myocardial infarction (STEMI)
B. Patients with cardiogenic shock or concomitant severe decompensated heart failure
C. Patients with myocardial infarction or stent thrombosis in spite of the maintenance of antiplatelet therapy
D. Restenosis in stented segments or previous sites of balloon angioplasty
2. Patients who cannot follow allocated DAPT schedule due to the planned surgery or elective procedure within 3 months after the stenting
3. Recent history of major surgery or evident events of gastrointestinal bleeding within 1 month from the procedure
4. Patients on anticoagulation therapy with warfarin or other anticoagulants
5. Life expectancy less than 1 year (such as malignancies or other chronic systemic diseases)
6. Pregnant women
7. Past history of allergy or other contraindications for the following medications/materials: aspirin, clopidogrel, prasugrel, ticagrelor, heparin, cobalt chromium, sirolimus

For peer review only

For peer review only

Additional files

Additional files 1: SPIRIT Checklist (PDF 95kb)

Additional file 2: SPIRIT Time schedule (PDF 105kb)

Additional file 3: Informed consent form (Korean version, PDF 209kb)

Additional file 4: IRB documentations

4-1: Seoul National University Hospital

4-2: SoonChunHyang University Cheonan Hospital

4-3: Hallym University Kangnam Sacred Heart Hospital

4-4: Korea University Guro Hospital

4-5: Ajou University Hospital

4-6: Chonnam National University Hospital

4-7: Kwangju Christian Hospital

4-8: Inje University Busan Paik Hospital

For peer review only

Figure 1. Study outline and randomization scheme

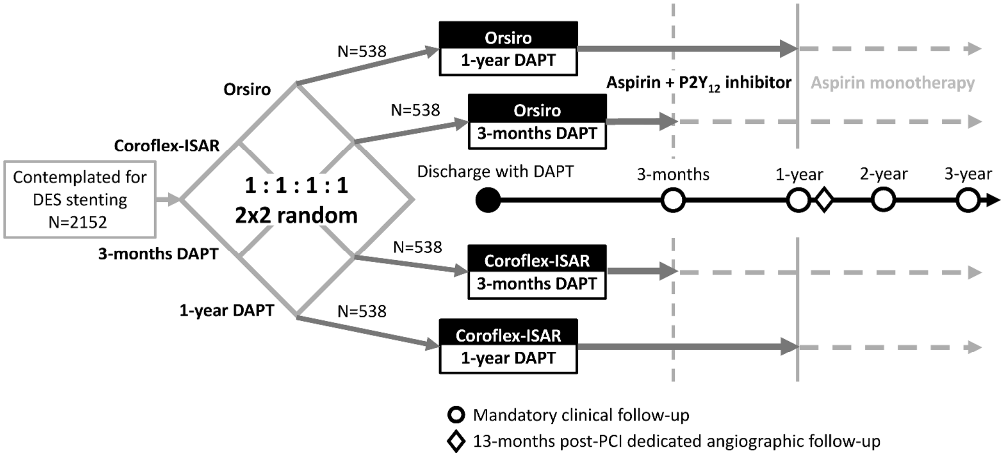

Figure 1. Study outline and randomization scheme

Appendix

Endpoint definitions

To gauge the clinical efficacy of each stent system, TLF as a composite of cardiac death, TVMI, and clinically driven TLR will be used as a surrogate for outcome measure. Though 1-year TLF rate will be presented as a primary endpoint of this trial, TLF and its components will be collected up to 3 years to delineate long-term clinical outcome. At the same time, NACE as a comprehensive outcome related to safety issue will be verified throughout the study period and it will disclose the consequences according to the two schemes of mandatory DAPT regimen. Definite or probable stent thrombosis and major bleeding as well as TLF will be regarded as components of NACE. Outcome measures and their components in this trial will follow the current data standards and regulatory guidelines [10 11].

1. Target lesion failure (TLF)

   The composite of clinically driven TLR, MI or cardiac death related to the target vessel. Target lesion is the segment where coronary intervention was performed, and the length of the target lesion is inclusive of the arterial segment treated with the stent and the 5mm proximal and 5mm distal to the treated section. Target vessel is the major native coronary artery or bypass graft containing the target lesion. A native coronary artery target vessel includes the arterial segments upstream and downstream from the target lesion and their major side branches. If it cannot be determined with certainty whether an MI or death is related to the target vessel, and at the same time if no other specific reasons can be given, it will be considered as a case of TLF.

2. Cardiac death

   Death by any cardiovascular mechanisms (arrhythmia, sudden death, low cardiac output heart failure, stroke, pulmonary embolism, or peripheral artery disease) will be

counted as a cardiac death. Unwitnessed death in a subject seen alive and clinically stable less than 24 hours before being found dead without any evidence supporting a specific non-cardiovascular cause of death will be also judged as a cardiac death. In addition, death caused by the immediate complications of the procedure will be managed as a cardiac death. Any death not covered by the above definitions, including death due to natural progression of underlying chronic disease, infection, accident, suicide or trauma will be handled as a non-cardiovascular death.

3. Target vessel myocardial infarction (TVMI)

   Among the case of acute spontaneous MI, TVMI is defined as a MI case with the evidence of myocardial necrosis in the vascular territory of previously treated target vessel. As well as direct evidence of invasive angiography, electrocardiographic or other imaging evidences such as echocardiography (e.g., newly developed regional wall motion abnormality or extension of previous abnormality) can be used to adjudicate the involvement of target vessel territory. Any types of MI related to stent thrombosis or restenosis of the target lesion will be included to TVMI case, but periprocedural MI (e.g., type 4a MI associated with and occurring within 48 hours of coronary intervention) and death with symptoms suggestive of myocardial ischemia but without direct evidence of target vessel involvement will be excluded from the outcome measure of TVMI.

4. Clinically driven target lesion revascularization (TLR)

   TLR indicates a revascularization procedure with repeated stenting, balloon angioplasty or surgical bypass grafting for restenosed or occluded culprit target lesion. TLR is clinically driven if the target lesion diameter stenosis is more than 50% by quantitative coronary angiography (QCA) and the subject has clinical or functional

For peer review only

ischemia that cannot be explained by another native coronary or bypass graft lesion. Even in the absence of ischemic symptoms or signs, TLR for a stented lesion with more than 70% diameter stenosis may be also considered clinically driven. And in case of the absence of QCA data or if a stenosis ≤50% is present, TLR may be considered clinically driven if severe ischemic signs and symptoms attributable to the target lesions are present. Meanwhile, repeated intervention or surgical bypass for any segment of a coronary artery containing a target lesion will be counted as a case of target vessel revascularization (TVR), and of course, TLR can be considered as TVR. In this context, target vessel failure (TVF) is defined as a composite of clinically driven TVR, cardiac death and TVMI, and this ancillary measure also will be counted throughout the study period.

5. Definite or probable stent thrombosis (ST)

Definite ST is defined as occurring when clinical presentation is consistent with acute coronary syndrome, and angiographic or pathologic examination with autopsy confirm stent occlusion or thrombus in the stented segment. Angiographic confirmation is the presence of a thrombus at the time of angiography that originates in or from the stent or in the segment 5mm proximal or distal to the stent. Probable ST is defined as any unexplained death occurring within the first 30 days that cannot be attributed to other causes. And irrespective of the time after the index procedure, any MI that is related to the territory of the implanted stent without angiographic confirmation of ST can be regarded as probable ST in the absence of any other obvious cause. Timing of the detection of ST will follow to the ARC (Academic Research Consortium) grading criteria: 0-24 hours after stent implantation is acute ST, >24 hours to 30 days after stenting is subacute ST, >30 days to 1 year after the procedure is late ST, and more than

For peer review only

1 year after stent implantation is very late ST.

6. Major bleeding

Type 3 or 5 bleeding according to the BARC (Bleeding Academic Research Consortium) criteria will be counted as a major bleeding event. Overt bleeding with hemoglobin drop more than 3 g/dL, any transfusion, procedure-related cardiac tamponade, bleeding requiring surgical intervention or intravenous vasoactive agents and intracranial or intraocular hemorrhage will be recorded as a major bleeding event. Fatal bleeding and subsequent death without no other explainable cause also can be categorized into this class of bleeding.

Stent platforms for this trial

Orsiro™ stent system	
Stent backbone	PRO-Kinetic Energy® stent system
Stent alloy	Cobalt chromium alloy
Passive coating	PROBIO™ amorphous silicon carbide coating
Active coating	BIOlute™ high molecular weight Poly-L-Lactic Acid (PLLA)
Coating dose of sirolimus	1.4µg/mm^2
Stent strut thickness	Nominal diameter 2.25~3.00mm: 60µm (0.0024")<br>3.50~4.00mm: 80µm (0.0031")
Nominal diameter	2.25 / 2.50 / 2.75 / 3.00 / 3.50 / 4.00mm
Nominal length	9 / 13 / 15 / 18 / 22 / 26 / 30 / 35 / 40mm
Lesion entry profile	0.017" (0.43mm)
Lesion crossing profile	0.039" (0.99 mm)
Nominal inflation pressure	8 atm
Rate burst pressure	16 atm

Coroflex ISAR™ stent system	
Stent backbone	CX Blue Ultra & Neo® stent system
Stent alloy	Cobalt chromium alloy
Coating dose of sirolimus	1.2µg/mm^2
Stent strut thickness	Nominal diameter 2.00~2.50mm (): 50µm (0.0020")<br>2.75~4.00mm (CX-Blue Neo): 60µm (0.0024")
Nominal diameter	2.00 / 2.25 / 2.50 / 2.75 / 3.00 / 3.50 / 4.00mm
Nominal length	2.00~2.50mm stent:

	9 / 14 / 16 / 19 / 24 / 27 / 32mm<br>2.75~4.00mm stent:<br>8 / 13 / 16 / 19 / 24 / 27 / 32mm
Lesion entry profile	0.016” (0.41mm)
Lesion crossing profile	0.031~0.037” (0.79~0.93mm)
Nominal inflation pressure	10 atm
Rate burst pressure	18 atm (15 atm in case of 4.00mm-sized stent)

For peer review only

HOST-IDEA 연구 대상자 설명문

서울대학교병원 순환기내과

연구 대상자 설명문 version: 1.1 (2016.2.3.)

1. 임상 연구 제목

관동맥 질환 치료의 최적의 조화로운 전략을 제시하기 위한 연구 - 차세대 약물 방출 스텐트 플랫폼을 이용한 관동맥 중재 시술과 단기간 이중 혈소판 억제 요법의 효용성 검증 (HOST-IDEA 연구)

2. 연구 책임자

서울대병원 순환기내과 교수 김효수

3. 임상 연구의 배경 및 목적

본 임상 연구는 최근 국내에 도입된 최신 관동맥 스텐트 (금속 그물망) 중의 하나인 Orsiro (오시로) 스텐트와 Coroflex ISAR (코로플랙스 아이사) 스텐트의 실제 임상 현장에서의 장단기 효용성과 안전성을 검증하고 스텐트 시술 이후 유지해야 하는 이중 혈소판 억제 요법의 적정 유지 기간에 대한 근거를 마련하기 위해 계획하였습니다.

차세대 약물 방출 스텐트 중의 하나인 Orsiro 스텐트는 스텐트 그물망 안으로 새살이 차오르는 것을 억제하는 약물이 관동맥 병변의 내벽에 모두 방출되어 그 역할을 다 하고 나면 약물을 머금고 있었던 스텐트 표면의 코팅 성분이 생체에 흡수되도록 설계한 스텐트입니다. 동시에 스텐트의 금속 가닥이 관동맥 내벽에 그대로 노출되지 않도록 아주 얇은 별도의 생체 적합성 코팅을 스텐트 가닥에 입혀, 약물을 머금은 코팅 성분이 그 역할을 다 한 이후에도 중합체 코팅 성분이 장기간 남아 있거나 스텐트 금속 가닥이 그대로 노출되어 있을 때 발생할 수 있는 관동맥 내벽의 만성 염증 등의 부작용을 최소화할 수 있을 것으로 보고 있습니다. 실제로도 동물 연구 및 인체를 대상으로 한 여러 임상 시험에서 생체 비적합 코팅 중합체나 스텐트 금속 가락이 동맥 조직에 만성적인 염증을 유발할 가능성을 대폭 개선시킨 결과를 보고하였습니다. 2014년에 해외 저명 의학 학술지 중 하나인 LANCET 지에 발표된 BIOSCIECNE 연구는 총 2119명을 대상

For peer review only - http://bmjopen.bmj.com/site/about/guidelines.xhtml

으로 기존에 널리 쓰이고 있는 에버롤리무스 용출 약물 스텐트와 Orsiro 스텐트의 임상 성적을 비교하였는데, 스텐트 시술 후 1년 시점에서 사망, 심근 경색, 혈관 재협착에 의한 재시술 등의 주요한 임상 성적에 있어 유의한 차이를 보이지 않았습니다. 이렇게 축적된 그간의 연구들을 바탕으로 Orsiro 스텐트는 세계 각국의 보건 당국으로부터 정식으로 시판 허가를 받아 최근 임상 현장에서 활발하게 쓰이고 있습니다.

Coroflex ISAR 스텐트는 Orsiro 스텐트와 같은 억제 약물을 사용하고 기존에 시판 중인 약물 방출 스텐트들 중 가장 가느다란 스텐트 금속 가락 두께를 자랑하는 스텐트입니다. 기존의 다른 약물 방출 스텐트들과는 달리 처음부터 스텐트 표면에 코팅 성분을 따로 입히지 않고 억제 약물을 일정 시간 동안 모두 다 용출하고 나면 스텐트의 금속 가락만 동맥 내벽에 남게 하여 코팅 성분에 의해 발생할지도 모르는 부작용 위험을 원천적으로 차단하는 것을 목표로 개발한 스텐트입니다. 그 안전성이 널리 입증된 단순 금속 스텐트의 장점을 취하면서도 약물 방출 스텐트의 효능은 겸비한 스텐트로 처음부터 따로 코팅 성분을 입히지 않는 대신, 억제 약물의 방출 속도를 적절히 조절해주는 또 다른 약물을 미량 첨가한 뒤 스텐트 금속 가락에 약물을 바로 입혀 스텐트의 치료 효과를 나타내게 됩니다. 코팅 성분을 사용하지 않지만, 여러 임상 시험에서 기존에 사용하고 있는 다른 약물 방출 스텐트와 비교하여 관동맥 질환 치료에 전혀 손색없는 성적을 보여주어 차세대 스텐트 중 하나로 기대를 모으고 있습니다. 일례로, 2011년에 역시 마찬가지로 해외 유수 의학 학술지 중의 하나인 CIRCULATION 지에 실린 ISAR-TEST 5 연구에서 총 3002명의 환자들을 대상으로 조타롤리무스 용출 스텐트와 Coroflex ISAR 스텐트의 임상 성적을 비교하였을 때 시술 후 1년 시점까지의 주요 임상 성적에 유의한 차이가 없음을 확인한 바 있습니다. 이들 근거를 바탕으로 Coroflex ISAR 스텐트는 최근 국내에도 정식으로 도입되어 실제 환자 치료에 쓰이고 있습니다.

그러나, 한정적인 조건을 만족시키는 관동맥 질환 환자들만을 대상으로 한 임상 시험과 달리 일상적인 임상 진료 현장에서 만나게 되는 다양한 관동맥 질환 환자들에게 Orsiro 스텐트와 Coroflex ISAR 스텐트를 사용하는 경우 치료 성적이 어떻게 나타날지 비교 연구를 통해 입증한 자료가 좀 더 필요하다고 할 수 있습니다. 더불어 스텐트의 장단기 안전성이 개선되면서 스텐트 시술 후 아스피린 및 클로피도그렐과 같은 항혈소판 약제를 이중으로 유지해야 하는 적정 기간을 기존에 최소 1년까지 권고하였던 것과 달리 환자의 상태가 안정적이라면 3개월까지만 짧게 사용해도 무방하다는 연구 결과들이 축적되고 있어, Orsiro 스텐트와 Coroflex ISAR 스텐트와 같은 차세대 스텐트에서도 이와 같은 권고를 할 수 있을지 관심이 모아지고 있습니다. 각각 2012년과 2014년 해외 유수 학술지에 발표된 RESET 연구(총 연구 대상 환자 수 2117명)와 OPTIMIZE 연구(총 연구 대상 환자 수 3119명)에서는 조타롤리무스 약물 방출 스텐트를 이용한

For peer review only - http://bmjopen.bmj.com/site/about/guidelines.xhtml

환자에서 이중 혈소판 억제 요법을 3개월까지만 유지한 군과 통상적인 12개월까지 유지한 군을 1:1로 비교 검정하였는데 심장 관련 사망, 심근 경색 재발, 주요한 출혈 합병증 등의 주요 임상 성적은 혈소판 억제 요법 유지 기간을 3개월까지 단축하여도 뚜렷한 차이가 없음을 입증한 바 있습니다. 최근에 개정된 유럽 진료 지침에서는 환자의 임상 상태가 안정적이라면 스텐트 시술 후 6개월까지만 이중 혈소판 억제 요법을 유지하고 이후에는 아스피린만 유지해도 무방하다는 임상 권고안이 채택된 바 있는데, 많은 전문가들은 앞으로 상태가 안정적인 상당 수 환자에서는 시술 후 3개월까지만 이중 혈소판 억제 요법을 유지해도 된다는 쪽으로 개정될 것이라 전망하고 있습니다. 이와 관련하여 스텐트의 효용성은 유지하면서 안전성은 개선시켜 우수한 임상 성적을 보이는 새로운 스텐트들이 도입되고 있는 이 때, 국내 환자들을 대상으로도 새로 도입된 차세대 스텐트를 적용할 때 어느 정도 기간까지 이중 혈소판 억제 요법을 유지해야 할지 그 근거 자료를 마련할 연구도 필요한 상황입니다. 본 연구는 3개월과 12개월의 이중 혈소판 억제 요법의 임상적 효용성을 비교할 예정으로, 진료 지침을 좀 더 전향적으로 개정하는데 도움이 될 귀중한 원천 자료를 제공해줄 수 있을 것으로 기대하고 있습니다.

이에 우리나라의 심혈관 질환 연구를 선도하고 있는 본 기관에서는 실제 임상 현장의 환자들에서 Orsiro 스텐트와 Coroflex ISAR 스텐트의 효용성과 안전성을 입증하면서 이중 혈소판 억제 요법의 적정 유지 기간에 대한 정보를 얻을 수 있도록 본 비교 연구를 계획하였습니다.

4. 임상 연구 참여 대상자 수 및 참여 기간

통계적 검정력 등을 고려한 본 임상 연구의 일차 등록 목표는 약 2152명입니다. 국내의 다른 유수 의료 기관과 합동으로 환자 등록을 진행하게 되며, 본 기관에서는 이 중 100명의 환자를 등록할 것을 계획하고 있습니다. 등록 마감 전까지 관동맥 스텐트 시술을 앞둔 환자 중 본 연구에 참여하기를 서면으로 동의한 환자라면 누구나 연구에 참여할 수 있습니다. 연구 참여에 동의하시면 스텐트 시술일로부터 최장 3년 시점까지의 경과를 추적 관찰하게 됩니다.

5. 임상 연구의 절차 및 방법

저희 병원에서 관동맥 질환 치료를 받으시고 연구 등록을 사전 동의하신 분들 중 심혈관 조영술을 시행하여 관동맥 질환을 확인하게 되면 스텐트 시술 시 Orsiro 스텐트와 Coroflex ISAR 스텐트 중 어떤 스텐트를 사용할지 인터넷 기반 무작위 배정 시스템을 통해 1:1로 배정되도록 결정하게 됩니다. 또한 동시에 이중 혈소판 억제 요법의 총 유지 기간을 통상적인 12개월과 이를

For peer review only - http://bmjopen.bmj.com/site/about/guidelines.xhtml

단축시킨 3개월의 두 가지 방법 중 한 가지로 결정하는 1:1 무작위 배정도 진행합니다. 때문에 무작위 배정을 하기 전까지는, 관동맥 질환의 진단을 위한 심혈관 조영술 검사를 시행하고 이에 대해 스텐트 시술을 하기 전에 미리 어떤 스텐트를 시술할지 결정하고 검사 및 시술을 진행할 수는 없습니다. 시술 시 사용하게 되는 스텐트 개수도 개별 환자의 병변 특성에 맞추어 결정하기 때문에 마찬가지로 미리 알거나 결정할 수 없습니다. 또한, 이중 혈소판 억제 요법을 언제까지 지속할 것인지에 대해서도 무작위 배정 전까지 미리 알거나 결정할 수 없습니다. 무작위 배정 시스템은 본 병원과 독립된 별도의 외부 기관 (CRScube사)에서 별도로 구별된 알고리즘으로 구성되어 있어 웹 시스템에 환자 등록 시 자동으로 연구 배정군이 결정되며, 환자 등록 과정이나 이후의 추적 과정에서 무작위 배정된 군을 임의로 변경할 수 없습니다. 다만, 시술 대상으로 결정한 관동맥 병변에 Orsiro 스텐트나 Coroflex ISAR 스텐트를 이용하여 스텐트 시술을 하게 되더라도 기존에 시판되어 널리 쓰이고 있는 다른 종류의 스텐트를 주요 시술 대상으로 선정한 병변 부위 이외의 관동맥 병변에 함께 사용하는 경우가 있을 수 있습니다. 또 실제 이중 항혈소판제 유지 요법의 준수 기간을 가급적 무작위 배정된 만큼 유지하는 것을 목표로 삼되, 환자의 상황에 따라 이후에 예정된 기간보다 좀 더 빨리 중단하거나 반대로 좀 더 오래 유지하게 되는 경우가 있을 수 있습니다.

연구 등록에 동의하고 스텐트 시술을 받으시면, 사전에 예정한 기간 동안 이중 혈소판 억제 요법을 유지하며 퇴원 후 1개월, 3개월, 1년, 2년, 3년 시점에 외래에서 경과 확인을 위한 추적 관찰을 시행하며, 일부 환자들은 첫 스텐트 시술을 받으신 이후 약 13개월 시점에 추적 관찰 검사를 위한 심혈관 조영술을 시행하게 됩니다. 13개월 시점의 심혈관 조영술 추적 검사와 첫 시술 후 1개월, 3개월, 1년, 2년, 3년 시점의 외래 추적 관찰은 심혈관계 질환으로 스텐트 시술을 받으시게 된 다른 환자들의 경우에도 통상적으로 시행하는 것으로서, 본 연구에 참여하시는 것으로 인해 불필요한 검사나 외래 방문 부담이 늘어나는 것은 아닙니다. 처음 Orsiro 스텐트나 Coroflex ISAR 스텐트를 삽입한 당시의 경과와 기본적인 혈액 검사 등의 임상 정보, 그리고 외래 방문 또는 13개월 시점의 심혈관 조영술을 위한 입원 시 경과를 기록하고 이를 향후 연구 분석을 위한 자료로 사용하게 됩니다. 연구에 등록 및 참여하시는 분들은 예정된 외래에 맞추어 저희 병원에 방문하시면 되고, 이에 대해서는 저희 연구진이 별도로 안내하여 도움을 드릴 것입니다.

6. 연구 대상자에게 예견되는 부작용, 위험과 불편함

본 연구에서는 관동맥 중재시술을 받으실 환자 중 본인의 의료 정보를 연구 목적으로 활용하는

For peer review only - http://bmjopen.bmj.com/site/about/guidelines.xhtml

것에 동의하는 분들만 등록하여 기본적인 임상 정보 및 시술과 관련된 자료를 수집합니다. 더불어 향후의 추적 관찰 기간 동안 발생할 수 있는 주요한 심혈관계 문제들과 이와 연관된 임상 성적이 두 가지 스텐트, 두 가지 이중 혈소판 억제 요법의 유지 기간 사이에 차이가 있는지 비교하게 됩니다. 실제 연구 기간 동안 특정 배정군에서 임상 성적에 약간의 차이가 발생할 가능성이 있지만, 본 연구진은 두 가지 스텐트의 임상 성적에 유의한 차이가 없고, 이중 혈소판 억제 요법의 유지 기간에 차등을 두어도 임상 성적에 역시 뚜렷한 차이가 없을 것으로 기대하고 있습니다.

이와 별개로, 본 연구에 참여할 때 통상적인 스텐트 시술을 받게 되는 다른 환자와 비교하여 추가적으로 예견되는 부작용이나 위험, 불편 사항은 없습니다. 환자분의 관동맥 질환 치료를 위해 시행하는 심혈관 조영술 및 중재술에 수반되는 일반적인 시술에 따른 위험이나 불편 사항은 본 연구에 참여하지 않더라도 경험하실 수 있는 것으로 여기에는 벽내 혈종, 곁가지 폐색, 스텐트 이동, 동맥 파열이나 천공, 박리(찢어짐), 색전증, 스텐트 가변형성, 뇌졸중, 조영제에 의한 급성 신기능 손상 등이 있을 수 있으며, 때로는 스텐트 시술을 시행한 병변에 대해 재시술을 진행해야 하는 경우도 생길 수 있습니다. 이들 부작용의 발생 위험은 일반적인 경우와 같으며 본 연구로 인해 특별히 증가하는 별도의 위험은 없습니다. 스텐트 시술을 받은 이후 3개월 또는 1년 여 동안 혈소판 기능을 억제하기 위해 아스피린과 클로피도그렐을 함께 복용함으로써 위장관 부작용 및 출혈, 발진, 두통, 현기증, 콜레스테롤 수치 증가, 백혈구감소증, 혈소판감소증 등의 문제가 생길 수 있습니다. 이중 혈소판 억제 요법은 심혈관 조영술 및 중재술 치료를 받는 모든 환자에서 일반적으로 적용되는 치료로 그 부작용의 빈도와 중증도는 대개 무시할 수 있는 수준에 그치는 경우가 많습니다. 그러나 본 연구에서는 이러한 부작용에 대한 모니터링도 함께 시행하여 환자 안전에 만전을 기할 것입니다.

7. 연구 대상자에게 예견되는 이득

본 연구에 참여하실 때 연구진에 포함된 연구간호사들에 의해 외래 및 입원 기간 동안 면밀한 추적 관찰을 받으실 수 있는 것 이외에는 별도의 금전적인 보상이나 비경제적 이점을 제공하지는 않습니다. 저희 연구진은 본 연구에 참여해주신 많은 분들에게서 얻은 소중한 자료를 토대로 Orsiro 스텐트와 Coroflex ISAR 스텐트의 효능을 확인하고 관동맥 질환 치료 후 이중 혈소판 억제 요법의 유지 기간에 차이를 두었을 때 발생할 수 있는 경과의 차이에 대해 좀 더 실제적인 정보를 얻어 연구에 참여하시는 분들은 물론이고, 다른 환자들께도 향후 더 나은 치료 대안을 제시하고자 합니다.

For peer review only - http://bmjopen.bmj.com/site/about/guidelines.xhtml

8. 연구 참여 비용 및 손실에 대한 보상

앞서 말씀 드렸듯이, 본 연구에서 사용되는 두 가지 스텐트는 국내 시판 허가를 이미 획득하여 여러 국내 유수의 병원들에서 실제 임상 현장에서 이미 쓰이고 있는 것들로, 미국과 유럽 등 다른 의료 선진국에서도 마찬가지로 널리 쓰이고 있는 스텐트입니다. 더욱이 본 연구에서 환자의 관동맥 병변에 대한 통상적인 치료 과정의 일부로 스텐트 시술이 진행되기 때문에, 연구에 등록하지 않고 통상적인 치료를 받는 경우와 비교하여 연구 참여자가 추가로 더 부담해야 하는 별도의 검사/처치/투약 사항은 없습니다. 즉, 저희 병원에서 관동맥 질환 치료를 받아오셨던 다른 환자들께서 부담하시는 정도 이외에 별도 비용 발생 소요가 없습니다. 때문에, 본 연구에서는 환자에게 스텐트 비용 및 치료에 필요한 시술 비용의 전부 또는 일부를 보전 및 지원하지 않습니다. 관동맥 병변이 있어 약물 용출 스텐트를 이용한 스텐트 시술이 필요한 환자에서 국내에 소개되어 활발하게 쓰이기 시작한 두 가지 새로운 스텐트 중 한가지 군에 무작위 배정하도록 하여 시술을 진행할 뿐, 본 연구에 참여하기 때문에 받지 않아도 될 스텐트 시술을 불필요하게 받게 된다거나, 실제 필요 병변보다 더 많은 관동맥 부위에 시술하지는 않습니다. Orsiro 스텐트와 Coroflex ISAR 스텐트 모두 국내 시판 중인 다른 스텐트들과 비슷한 수준에서 보험 수가가 책정되어 있으며 (Orsiro 스텐트 : 비보험 일반 단가 개당 2,210,750원, 보험 단가 개당 1,842,294원, Coroflex ISAR 스텐트 : 비보험 일반 단가 개당 2,199,600원, 보험 단가 개당 1,833,000원), 시술 재료대와 시술 비용에 차이가 거의 없습니다.

그러나, 이런 스텐트 수가를 환자가 모두 부담하는 것은 아닙니다. 다른 스텐트를 이용한 시술의 경우와 마찬가지로 이 두 가지 스텐트를 이용한 시술도 모두 의료 보험 적용을 받기에 불필요한 의료 비용의 상승을 초래하지 않습니다. 2014년 12월부터 보건복지부가 심장 스텐트의 건강 보험 적용 개수 제한을 기존 3개까지 적용하던 것을 없애고 4개 이상의 스텐트를 시술 받는 환자도 개당 9만원 정도(보험 단가의 5%에 해당)만 부담하도록 건강 보험 적용을 확대하였기 때문에 스텐트 시술 시 실질적인 재료비 부담이 크게 줄어들었습니다. 참고로, 2013년에 저희 병원을 포함하여 국내 유수의 기관들이 주축이 된 EXCELLENT/RESOLUTE-Korea 스텐트 등록 연구의 결과를 미국 심장 내과 학회지(JACC)에 발표했던 결과를 살펴보면 2개 이상 복수의 혈관에 스텐트를 사용한 환자의 비율이 대략 30% 정도에 지나지 않습니다. 또한 환자 1인 당 사용한 스텐트의 평균 개수가 1.5개 정도 밖에 되지 않았기 때문에 본 연구에 참여하게 되어 발생하는 스텐트 비용은 본 연구에 참여하지 않고 통상적인 스텐트 시술을 받는 경우와 비교하여 차이가 거의 없을 것으로 예상하고 있습니다. 두 스텐트의 가격이 서로 비슷하고 보험 적용에

For peer review only - http://bmjopen.bmj.com/site/about/guidelines.xhtml

있어서도 특별한 구분은 없기에 배정된 연구군에 따른 비용 차이도 거의 없을 것입니다. 다만 이중 혈소판제 유지 기간에 따른 약제비에 약간의 차이가 발생할 수 있습니다. 3개월 DAPT 군에 배정된 환자의 경우 통상적인 6개월~1년까지 DAPT를 유지하는 경우와 비교하여 3개월 DAPT 군에 배정된 환자가 얻게 될 경제적 유인이 있다고 볼 수도 있지만, 다양한 형태의 저렴한 항혈소판제 복제약이 출시되고 이들 약제비에 대한 건강보험 적용도 더욱 확대되면서 그 부담이 전보다 크게 줄어들어 그 차이는 실제로 미미한 수준입니다. 본 연구에 참여하지 않는 다른 환자들의 경우에도 스텐트 시술 후 사용해야 하는 약제비에 대해 실제적으로 느끼는 부담이 예전보다 상당히 줄었다고 하시는 경우가 많은 실정입니다.

이처럼, 본 연구에 참여하신다고 하여 실질적으로는 별도의 추가 비용이 발생하지 않습니다. 그리고 입원이나 외래 추적 관찰 시 시행하는 각종 혈액 검사, 심전도나 방사선 검사 등의 제반 검사는 본 연구에 참여하지 않는 일반적인 다른 환자들 모두에서 시행하는 검사로서, 본 연구만을 위해 별도의 추가 검사를 시행하지는 않습니다. 따라서 앞서 말씀 드린 바와 같이, 본 연구에 참여하시는 것에 대해 따로 금전적 보상을 해드리지는 않습니다. 다만, 연구 책임자를 비롯한 연구진 모두는 본 연구에 참여한 환자들 중, 연구 계획을 충실히 이행한 상황에서 Orsiro 스텐트와 Coroflex ISAR 스텐트에 의해 직접적이고 명백한 인과 관계 하에 초래된 유해 사례 발생 시, 이에 대한 치료 또는 입원이 필요하게 되는 경우 환자의 부담이 최소화되도록 노력할 것입니다. 이중 혈소판 억제 요법의 차이에 따라 발생할 수 있는 잠재적인 합병증 발생 위험에 대해서도 마찬가지로 환자의 부담이 최소화되도록 노력할 것입니다. 하지만 연구 책임자의 후원 하에 집행되지 않았거나 제공하지 않은 의약품이나 치료 재료 등으로 인해 발생한 유해 사례 및 이에 수반한 손상이 있는 경우에 대해서는 책임을 지지 않습니다. 또, 임상 연구에 의한 효과 또는 혜택을 제공하지 못한 것에 대한 보상이나 서로 합의한 연구 계획을 이행하지 않음으로 야기된 손상이나 이차적인 문제들이 있는 경우, 연구의 유의성과 안전에 영향을 미칠 수 있는 계획 준수 위반 사항이 있는 경우, 피험자의 부주의에서 초래된 손상이 발생하는 경우, 질병의 자연 경과에 의해 야기된 손상의 경우에 대해서도 보상하지 않습니다. 지금까지 말씀 드린 본 연구의 피해 보상에 대한 규약을 환자 설명문 말미에서 다시 한 번 별도로 정리하였습니다.

9. 자발적 참여 및 동의 철회

본 연구는 사전에 연구에 대해 충분한 설명을 듣고 자발적인 의지로 연구 참여 의사를 밝힌 분들에 한해 등록을 진행합니다. 때문에 연구에 대한 설명을 들으신 이후에도 참여 의사가 없으시다면 아무런 불이익 없이 연구 등록을 거부할 수 있습니다. 관동맥 질환에 대해 Orsiro

For peer review only - http://bmjopen.bmj.com/site/about/guidelines.xhtml

스텐트나 Coroflex ISAR 스텐트를 이용한 중재 시술을 받으신 분들이라 하여 반드시 이 연구에 참여해야 하는 의무를 지는 것은 아닙니다. 또한, 연구 참여에 동의하여 계획한 연구 일정이 진행되는 중에라도 연구 참여를 철회하고자 하는 의사가 있으면 언제든지 아무런 불이익이나 차별 없이 연구 참여를 철회할 수 있습니다. 도중에 연구 참여 의사를 철회하신 분이라 하더라도, 연구에 처음부터 참여하지 않으셨던 다른 환자들과 마찬가지로 일반적인 진료 지침에 의거한 심혈관 질환에 대한 통상적인 치료를 본 서울대병원 순환기내과에서 계속해서 받으실 수 있습니다. 연구가 진행되는 도중 지속적인 연구 참여 의지에 영향을 줄 수 있는 새로운 정보를 얻게 되는 경우 연구진은 적시에 연구 참여 대상자 본인 또는 그 대리인에게 제반 정보를 제공할 것입니다.

연구에 참여하지 않기로 결정하신다면, 1) Orsiro/Coroflex ISAR 스텐트를 제외한 기존에 시판 중인 다른 스텐트를 이용하여 스텐트 시술을 진행하고, 2) 3개월 또는 1년의 이중 혈소판 억제 요법 유지 기간에 구애 받지 않고 통상적인 진료 지침대로 기존에 다른 환자분들이 받고 계신 것과 같이 최소 6개월 또는 1년까지 이중 혈소판 억제 요법을 유지하게 됩니다. 현행 진료 지침대로 통상적인 진단/검사/시술이 이뤄지기 때문에 본 연구에 참여하지 않는다 하여 특별한 불이익이 발생하지는 않을 것입니다.

10. 개인 정보 보호 및 개인 정보 제공에 관한 사항

본 연구에서는 연구 참여 대상자의 영문 이니셜과 나이를 제외한 병록 번호나 주민등록번호, 주소지 등 신상을 파악할 수 있는 여타의 개인 정보는 원천적으로 수집하지 않습니다. 또한 연구를 위해 수집한 임상 정보나 시술 관련 사항은 연구 결과가 출판된 이후까지 모두 비밀로 기록하고 보호할 것입니다. 다만, 임상 연구의 모니터 요원이나 제반 사항을 점검하는 사람, 서울대학교병원 의학연구윤리심의위원회 (피험자보호센터) 및 보건복지부 장관이 관계 법령에 따라 연구의 절차와 자료의 품질을 검증하기 위하여 지정한 사람은 대상자의 신상에 관한 비밀을 보호하고 이를 침해하지 않는 범위에서 대상자의 연구 기록을 열람할 수 있습니다. 연구 참여 의향을 밝힌 연구 대상자 또는 대상자의 대리인이 연구 참여 동의서에 서명하는 경우 이러한 자료의 열람이 제한적인 경우에 허용된다는 사실을 미리 알고 이에 대해서도 동의하여 이들 자료의 열람을 허용한 것으로 보게 됩니다.

11. 담당자 연락처

For peer review only - http://bmjopen.bmj.com/site/about/guidelines.xhtml

본 임상 연구에 참여하시는 도중 문제가 발생하거나 본 연구에 대해 질문이 있으신 경우 다음 연구 담당자에게 연락하십시오.

서울대학교병원 순환기내과

연구 책임자	이름 : 김효수	전화 번호 : 02-2072-1973
담당 연구 코디네이터	이름 :	전화 번호 : 02-2072-4034

또한, 연구 대상자의 권익에 대한 문제, 우려, 질문이 있을 때에는 서울대학교병원 의학윤리심의위원회 (02-2072-0694) 또는 임상연구윤리센터 (02-2072-3509)에 연락하여 도움을 받으실 수도 있습니다.

For peer review only

For peer review only - http://bmjopen.bmj.com/site/about/guidelines.xhtml

HOST-IDEA 연구 피해 보상에 대한 규약

본 연구자 주도 임상시험의 의뢰자인 김효수는 임상시험 기간 동안 피험자에게 발생하는 피해에 관하여 아래의 원칙에 따라 책임을 진다.

1. 보상 원칙

- 피험자의 신체적인 손상(사망 포함)에 대해 보상한다.
- 손상의 원인이 임상시험 참여로 인해 발생하였을 때 피험자에게 보상한다.

2. 비보상 원칙

- 본 임상시험의 후원 하에 집행되지 않았거나 본 임상시험에서 제공하지 않은 의약품/치료 재료로 발생한 이상 반응에 의한 손상
- 임상시험용 의약품/치료 재료를 사용함에 있어 당국의 허가 과정에서 보고된 의약품/치료 재료 사용 시의 치료 효과 또는 혜택이 실제 개개 환자에서 제대로 충분히 달성되지 않을 수 있는 잠재적 위험과 관련된 보상
- 서로 합의한 임상시험 계획에서 이탈하고 계약 준수 의무를 위반하여 야기된 손상이나 이차적인 문제가 발생하는 경우
- 피험자의 부주의에서 초래된 손상
- 질병의 자연 경과에 의해 야기된 손상

3. 보상 평가 기준

보상 수준은 손상의 본질과 정도, 지속성 여부 등에 상응하는 적절한 정도여야 하며, 유사한 손상 또는 손실에 대해 대한민국 법정에 의해 기존 판시된 일반적인 보상 수준과 동일해야 한다. 이러한 보상 수준에 대해서 피험자와 의뢰자 사이에 이견이 있을 경우, 양자가 수용할 수 있는 전문가나 독립적인 별도의 중재기관의 자문을 통해 보상 수준을 결정하고 이에 따라 보상한다.

본 연구자 주도 임상시험의 의뢰자인 김효수는 본 임상시험에서 피험자가 입은 피해에 대하여 상기 내용에 의거하여 책임을 질 것을 서약합니다.

서울대학교병원 순환기내과 김 효 수 (인)

For peer review only - http://bmjopen.bmj.com/site/about/guidelines.xhtml

HOST-IDEA 연구 대상자 동의서

1. 본인은 임상연구에 대해 구두로 설명을 받고 상기 연구 설명문을 읽었으며 담당 연구원과 이 연구에 대하여 충분히 의논하였습니다.

2. 본인은 연구의 위험과 이득에 관하여 들었으며 나의 질문에 만족할 만한 답변을 얻었습니다.

3. 본인은 이 연구에 참여하는 것에 대하여 자발적으로 동의합니다.

4. 본인은 이후의 치료에 영향을 받지 않고 언제든지 연구의 참여를 거부하거나 연구의 참여를 중도에 철회할 수 있고 이러한 결정이 나에게 어떠한 해가 되지 않을 것이라는 것을 알고 있습니다.

5. 본인은 이 설명서 및 동의서에 서명함으로써 의학 연구 목적으로 나의 개인정보가 현행 법률과 규정이 허용하는 범위 내에서 연구자가 수집하고 처리하는데 동의합니다.

6. 본인은 연구 설명문 및 동의서의 사본을 받을 것을 알고 있습니다.

연구대상자 성명	서명	날짜(년/월/일)
시험자/연구자 성명	서명	날짜(년/월/일)
법정대리인 성명	서명	날짜(년/월/일)
(대상자와 대리인과의 관계)		

For peer review only - http://bmjopen.bmj.com/site/about/guidelines.xhtml

Figure. Time schedule of enrollment, interventions, and assessment for participants

			STUDY PERIOD					
	Enrolment	Allocation	Post-allocation					Close-out
**TIMEPOINT****	*Day-1 to 0*	Day 0	*Mo. 1*	*Mo. 3*	*Yr. 1*	*Mo. 13*	*Yr 2.*	*Yr. 3*
**ENROLMENT:**								
Eligibility screen	X							
Informed consent	X							
Demographic details	X							
Medical history	X							
Allocation		X						
**INTERVENTIONS:**								
Stent type **Orsiro vs. CX-ISAR**			X					
DAPT maintenance **3 mo. Vs. 12 mo.**								
**ASSESSMENTS:**								
Anthropometric measurements and body compositions		X	X	X	X	X	X	X
Blood samples		X	X	X	X	X	X.	X
Cardiac function assessed by echocardiography		X			X		X	X
Angiographic follow-up						X*		
Compliance for DAPT			X	X	X			
Events and clinical outcomes			X	X	X	X	X	X
Adverse reactions			X	X	X	X	X	X

* Angiographic follow-up is not mandatory for every participant. Details for angiographic surveillance will follow the policy of each participating center.

For peer review only

STANDARD PROTOCOL ITEMS: RECOMMENDATIONS FOR INTERVENTIONAL TRIALS

SPIRIT 2013 Checklist: Recommended items to address in a clinical trial protocol and related documents*

Section/item	Item No	Description	Addressed on page number
**Administrative information**			
Title	1	Descriptive title identifying the study design, population, interventions, and, if applicable, trial acronym	______1______
Trial registration	2a	Trial identifier and registry name. If not yet registered, name of intended registry	______4______
	2b	All items from the World Health Organization Trial Registration Data Set	_____NA______
Protocol version	3	Date and version identifier	______22_____
Funding	4	Sources and types of financial, material, and other support	____23-24_____
Roles and responsibilities	5a	Names, affiliations, and roles of protocol contributors	_____24______
	5b	Name and contact information for the trial sponsor	_____23______
	5c	Role of study sponsor and funders, if any, in study design; collection, management, analysis, and interpretation of data; writing of the report; and the decision to submit the report for publication, including whether they will have ultimate authority over any of these activities	_____23-24____
	5d	Composition, roles, and responsibilities of the coordinating centre, steering committee, endpoint adjudication committee, data management team, and other individuals or groups overseeing the trial, if applicable (see Item 21a for data monitoring committee)	_____NA______

For peer review only

**Introduction**			
Background and rationale	6a	Description of research question and justification for undertaking the trial, including summary of relevant studies (published and unpublished) examining benefits and harms for each intervention	_____5-7_____
	6b	Explanation for choice of comparators	_____5-7_____
Objectives	7	Specific objectives or hypotheses	_____7_______
Trial design	8	Description of trial design including type of trial (eg, parallel group, crossover, factorial, single group), allocation ratio, and framework (eg, superiority, equivalence, noninferiority, exploratory)	_____7_______
**Methods: Participants, interventions, and outcomes**			
Study setting	9	Description of study settings (eg, community clinic, academic hospital) and list of countries where data will be collected. Reference to where list of study sites can be obtained	_____8_______
Eligibility criteria	10	Inclusion and exclusion criteria for participants. If applicable, eligibility criteria for study centres and individuals who will perform the interventions (eg, surgeons, psychotherapists)	_____8-9_____
Interventions	11a	Interventions for each group with sufficient detail to allow replication, including how and when they will be administered	_____12-13___
	11b	Criteria for discontinuing or modifying allocated interventions for a given trial participant (eg, drug dose change in response to harms, participant request, or improving/worsening disease)	_____13-14___
	11c	Strategies to improve adherence to intervention protocols, and any procedures for monitoring adherence (eg, drug tablet return, laboratory tests)	_____13-14___
	11d	Relevant concomitant care and interventions that are permitted or prohibited during the trial	_____13-14___
Outcomes	12	Primary, secondary, and other outcomes, including the specific measurement variable (eg, systolic blood pressure), analysis metric (eg, change from baseline, final value, time to event), method of aggregation (eg, median, proportion), and time point for each outcome. Explanation of the clinical relevance of chosen efficacy and harm outcomes is strongly recommended	_____15______
Participant timeline	13	Time schedule of enrolment, interventions (including any run-ins and washouts), assessments, and visits for participants. A schematic diagram is highly recommended (see Figure)	______12_____

For peer review only - http://bmjopen.bmj.com/site/about/guidelines.xhtml

Sample size	14	Estimated number of participants needed to achieve study objectives and how it was determined, including clinical and statistical assumptions supporting any sample size calculations	____10-12____
Recruitment	15	Strategies for achieving adequate participant enrolment to reach target sample size	_____13-14___

Methods: Assignment of interventions (for controlled trials)

Allocation:

Sequence generation	16a	Method of generating the allocation sequence (eg, computer-generated random numbers), and list of any factors for stratification. To reduce predictability of a random sequence, details of any planned restriction (eg, blocking) should be provided in a separate document that is unavailable to those who enrol participants or assign interventions	______12_____
Allocation concealment mechanism	16b	Mechanism of implementing the allocation sequence (eg, central telephone; sequentially numbered, opaque, sealed envelopes), describing any steps to conceal the sequence until interventions are assigned	______12_____
Implementation	16c	Who will generate the allocation sequence, who will enrol participants, and who will assign participants to interventions	______12_____
Blinding (masking)	17a	Who will be blinded after assignment to interventions (eg, trial participants, care providers, outcome assessors, data analysts), and how	______12_____
	17b	If blinded, circumstances under which unblinding is permissible, and procedure for revealing a participant’s allocated intervention during the trial	______NA_____

Methods: Data collection, management, and analysis

Data collection methods	18a	Plans for assessment and collection of outcome, baseline, and other trial data, including any related processes to promote data quality (eg, duplicate measurements, training of assessors) and a description of study instruments (eg, questionnaires, laboratory tests) along with their reliability and validity, if known. Reference to where data collection forms can be found, if not in the protocol	______14_____
	18b	Plans to promote participant retention and complete follow-up, including list of any outcome data to be collected for participants who discontinue or deviate from intervention protocols	______14_____

For peer review only - http://bmjopen.bmj.com/site/about/guidelines.xhtml

Data management	19	Plans for data entry, coding, security, and storage, including any related processes to promote data quality (eg, double data entry; range checks for data values). Reference to where details of data management procedures can be found, if not in the protocol	______14_____
Statistical methods	20a	Statistical methods for analysing primary and secondary outcomes. Reference to where other details of the statistical analysis plan can be found, if not in the protocol	______15_____
	20b	Methods for any additional analyses (eg, subgroup and adjusted analyses)	______15_____
	20c	Definition of analysis population relating to protocol non-adherence (eg, as randomised analysis), and any statistical methods to handle missing data (eg, multiple imputation)	______15_____
**Methods: Monitoring**			
Data monitoring	21a	Composition of data monitoring committee (DMC); summary of its role and reporting structure; statement of whether it is independent from the sponsor and competing interests; and reference to where further details about its charter can be found, if not in the protocol. Alternatively, an explanation of why a DMC is not needed	______14_____
	21b	Description of any interim analyses and stopping guidelines, including who will have access to these interim results and make the final decision to terminate the trial	______14_____
Harms	22	Plans for collecting, assessing, reporting, and managing solicited and spontaneously reported adverse events and other unintended effects of trial interventions or trial conduct	______14_____
Auditing	23	Frequency and procedures for auditing trial conduct, if any, and whether the process will be independent from investigators and the sponsor	______14_____
**Ethics and dissemination**			
Research ethics approval	24	Plans for seeking research ethics committee/institutional review board (REC/IRB) approval	______22_____
Protocol amendments	25	Plans for communicating important protocol modifications (eg, changes to eligibility criteria, outcomes, analyses) to relevant parties (eg, investigators, REC/IRBs, trial participants, trial registries, journals, regulators)	______23_____

For peer review only - http://bmjopen.bmj.com/site/about/guidelines.xhtml

Consent or assent	26a	Who will obtain informed consent or assent from potential trial participants or authorised surrogates, and how (see Item 32)	______22_____
	26b	Additional consent provisions for collection and use of participant data and biological specimens in ancillary studies, if applicable	______NA_____
Confidentiality	27	How personal information about potential and enrolled participants will be collected, shared, and maintained in order to protect confidentiality before, during, and after the trial	_____12, 22___
Declaration of interests	28	Financial and other competing interests for principal investigators for the overall trial and each study site	_____24, 25___
Access to data	29	Statement of who will have access to the final trial dataset, and disclosure of contractual agreements that limit such access for investigators	_____24, 25___
Ancillary and post-trial care	30	Provisions, if any, for ancillary and post-trial care, and for compensation to those who suffer harm from trial participation	_____NA_____
Dissemination policy	31a	Plans for investigators and sponsor to communicate trial results to participants, healthcare professionals, the public, and other relevant groups (eg, via publication, reporting in results databases, or other data sharing arrangements), including any publication restrictions	______25_____
	31b	Authorship eligibility guidelines and any intended use of professional writers	______25_____
	31c	Plans, if any, for granting public access to the full protocol, participant-level dataset, and statistical code	______NA_____
**Appendices**			
Informed consent materials	32	Model consent form and other related documentation given to participants and authorised surrogates	__supplement__
Biological specimens	33	Plans for collection, laboratory evaluation, and storage of biological specimens for genetic or molecular analysis in the current trial and for future use in ancillary studies, if applicable	______NA_____

*It is strongly recommended that this checklist be read in conjunction with the SPIRIT 2013 Explanation & Elaboration for important clarification on the items. Amendments to the protocol should be tracked and dated. The SPIRIT checklist is copyrighted by the SPIRIT Group under the Creative Commons "Attribution-NonCommercial-NoDerivs 3.0 Unported" license.

For peer review only
